# Supplementary material for: Point-of-care lung ultrasonography for early identification of mild COVID-19: a prospective cohort of outpatients in a Swiss screening center
Source: BMJ Open. 2022 Jun 23;12(6):e060181. doi: 10.1136/bmjopen-2021-060181 (PMC9234434; doi:10.1136/bmjopen-2021-060181)
Supplement: Supplementary data [file bmjopen-2021-060181supp001.pdf]

BMJ Publishing Group Limited (BMJ) disclaims all liability and responsibility arising from any reliance placed on this supplemental material which has been supplied by the author(s)

Supplementary Tables.

Supplementary Table 1. Characteristics of study participants comparing healthy controls and patients with a lower respiratory tract infection (COVID^{pos} and COVID^{neg}).

	**All (n=178)**	**LRTI patients**	**Control patients**	
Female sex	112 (63)	84 (63)	28 (64)	0.910
Age, years; Median (IQR)	34 [28, 45]	35 [29, 46]	31 [25, 42]	0.007
Pulmonary disease [a]	3 (1.7)	3 (2.2)	0 (0)	0.317
Current cigarettes smoker	51 (29)	39 (29)	12 (27)	0.816

Data are presented as n (%) unless otherwise indicated.
Missing values: 0
Abbreviations: IQR, interquartile range; LRTI, Lower respiratory tract infection
[a] COPD, fibrosis.

Supplementary Table 2. Lung ultrasound characteristics of study participants comparing healthy controls and patients with a lower respiratory tract infection (COVID^{pos} and COVID^{neg}).

	**All (n=178)**	**LRTI patients (n=134)**	**Control patients (n=44)**	
Abnormal lung ultrasound	45 (25)	41 (31)	4 (9.1)	0.004
Abnormal lung ultrasound apart from focal B lines	31 (17)	30 (22)	1 (2.2)	0.002
Multifocal	16 (9.0)	16 (12)	0 (0)	0.016
Bilateral	8 (4.5)	8 (6.0)	0 (0)	0.097
Number of pathologic zones; Median (IQR)	0 [0, 0.7]	0 [0, 1]	0 [0, 0]	0.003
Pathologic B lines (≥3)	23 (13)	20 (15)	3 (6.8)	0.164
Confluent B lines (White lung)	12 (6.7)	11 (8.2)	1 (2.3)	0.173
Thickening of the pleura with pleural line irregularities	18 (10)	18 (13)	0 (0)	0.010
Consolidations (>1cm)	1 (0.6)	1 (0.8)	0 (0)	0.566
Pleural effusion	0 (0)	0 (0)	0 (0)	
LUS score; Median (IQR)	0 [0, 0.75]	0 [0, 1]	0 [0, 0]	0.003

Data are presented as n (%) unless otherwise indicated.
Abbreviations: IQR, interquartile range.

Supplementary Table 3. Lung ultrasound characteristics of study participants comparing healthy controls and COVID-19 patients

	**All (n=75)**	**COVID-19 patients (n = 31)**	**Control patients (n = 44)**	
Abnormal lung ultrasound	18 (24.0)	14 (45)	4 (9.1)	0.001
Abnormal lung ultrasound apart from focal B lines	10 (13)	9 (29)	1 (2.2)	0.003
Multifocal	6 (8)	6 (19)	0 (0)	0.009
Bilateral	3 (4)	3 (9.7)	0 (0)	0.132
Number of pathologic zones; Median (IQR)	0 [0, 0]	0 [0, 1]	0 [0, 0]	< 0.001
Pathologic B lines (≥3)	9 (12)	6 (19)	3 (6.8)	0.199
Confluent B lines (White lung)	5 (6.7)	4 (13)	1 (2.3)	0.178
Thickening of the pleura with pleural line irregularities	6 (8)	6 (19)	0 (0.0)	0.009
Consolidations (>1cm)	0 (0)	0 (0)	0 (0)	
Pleural effusion	0 (0)	0 (0)	0 (0)	
LUS score; Median (IQR)	0 [0, 0]	0 [0, 2.5]	0 [0, 0]	<0.001

Data are presented as n (%) unless otherwise indicated.
Abbreviations: IQR, interquartile range.
