# Supplementary material for: Point-of-care lung ultrasonography for early identification of mild COVID-19: a prospective cohort of outpatients in a Swiss screening center
Source: BMJ Open. 2022 Jun 23;12(6):e060181. doi: 10.1136/bmjopen-2021-060181 (PMC9234434; doi:10.1136/bmjopen-2021-060181)
Supplement: Author's manuscript [file bmjopen-2021-060181.draft_revisions.pdf]

BMJ Open

BMJ Open is committed to open peer review. As part of this commitment we make the peer review history of every article we publish publicly available.

When an article is published we post the peer reviewers' comments and the authors' responses online. We also post the versions of the paper that were used during peer review. These are the versions that the peer review comments apply to.

The versions of the paper that follow are the versions that were submitted during the peer review process. They are not the versions of record or the final published versions. They should not be cited or distributed as the published version of this manuscript.

BMJ Open is an open access journal and the full, final, typeset and author-corrected version of record of the manuscript is available on our site with no access controls, subscription charges or pay-per-view fees (http://bmjopen.bmj.com).

If you have any questions on BMJ Open's open peer review process please email info.bmjopen@bmj.com

Point-of-care lung ultrasonography for early identification of mild COVID-19: a prospective cohort of outpatients in a Swiss screening center

Journal:	*BMJ Open*
Manuscript ID	bmjopen-2021-060181
Article Type:	Original research
Date Submitted by the Author:	14-Dec-2021

Complete List of Authors:	Schaad, Siméon; Lausanne University Hospital<br>Brahier, Thomas; Lausanne University Hospital<br>HARTLEY, Mary-Anne; University of Lausanne, Digital global Health Department; EPFL<br>CORDONNIER, Jean-Baptiste; EPFL<br>BOSSO, Luca; Lausanne University Hospital Emergency Care Service, Emergency Department<br>ESPEJO, Tanguy; Lausanne University Hospital Emergency Care Service, Emergency Department<br>PANTET, Olivier; Lausanne University Hospital Adult Intensive Care Unit<br>Hugli, Olivier; University Hospital of Lausanne, Emergency department<br>Carron, Pierre-Nicolas; Centre Hospitalier Universitaire Vaudois, Emergency<br>MEUWLY, Jean-Yves; Lausanne University Hospital Division of Radio-diagnostics and Interventional Radiology, Department of Radiology<br>Boillat-Blanco, Noémie; Infectious Diseases Service

Keywords:	COVID-19, ULTRASONOGRAPHY, Diagnostic radiology < RADIOLOGY & IMAGING, INFECTIOUS DISEASES

SCHOLARONE™
Manuscripts

I, the Submitting Author has the right to grant and does grant on behalf of all authors of the Work (as defined in the below author licence), an exclusive licence and/or a non-exclusive licence for contributions from authors who are: i) UK Crown employees; ii) where BMJ has agreed a CC-BY licence shall apply, and/or iii) in accordance with the terms applicable for US Federal Government officers or employees acting as part of their official duties; on a worldwide, perpetual, irrevocable, royalty-free basis to BMJ Publishing Group Ltd ("BMJ") its licensees and where the relevant Journal is co-owned by BMJ to the co-owners of the Journal, to publish the Work in this journal and any other BMJ products and to exploit all rights, as set out in our licence.

The Submitting Author accepts and understands that any supply made under these terms is made by BMJ to the Submitting Author unless you are acting as an employee on behalf of your employer or a postgraduate student of an affiliated institution which is paying any applicable article publishing charge ("APC") for Open Access articles. Where the Submitting Author wishes to make the Work available on an Open Access basis (and intends to pay the relevant APC), the terms of reuse of such Open Access shall be governed by a Creative Commons licence – details of these licences and which Creative Commons licence will apply to this Work are set out in our licence referred to above.

Other than as permitted in any relevant BMJ Author's Self Archiving Policies, I confirm this Work has not been accepted for publication elsewhere, is not being considered for publication elsewhere and does not duplicate material already published. I confirm all authors consent to publication of this Work and authorise the granting of this licence.

For peer review only

Point-of-care lung ultrasonography for early identification of mild COVID-19: a prospective cohort of outpatients in a Swiss screening center

Siméon SCHAAD[1*†], Thomas BRAHIER[1*], Mary-Anne HARTLEY[2,3], Jean-Baptiste CORDONNIER[3], Luca BOSSO[5], Tanguy ESPEJO[5], Olivier PANTET[4], Olivier HUGLI[5], Pierre-Nicolas CARRON[5], Jean-Yves MEUWLY[6*], Noémie BOILLAT-BLANCO[1*]

*Equal contribution to this work

†Corresponding author

Key words: COVID-19, lung ultrasound, screening, outpatients

[1] Infectious Diseases Service, University Hospital of Lausanne, Switzerland; [2] Digital global Health Department, Center for primary care and public health, University of Lausanne, Switzerland; [3] Machine Learning and Optimization Laboratory, EPFL, Switzerland; [4] Intensive Care Unit, University Hospital of Lausanne, Switzerland; [5] Emergency Department, University Hospital of Lausanne, Switzerland; [6] Department of Radiology, University Hospital of Lausanne, Switzerland.

Contact information: Siméon Schaad, Service of Infectious Diseases, University Hospital of Lausanne (CHUV), Rue du Bugnon 46, 1011 Lausanne, Switzerland, Phone: +41 79 524 15 85, E-mail: simeon.schaad@unil.ch

Word count : 3435

Funding and support: this work was supported by the Leenards foundation and by Lausanne University Hospital

For peer review only

Abstract

Objectives

Early identification of SARS-CoV-2 infection is important to guide quarantine and reduce transmission. This study evaluates the diagnostic performance of lung ultrasound (LUS), an affordable, consumable-free point-of-care tool, for COVID-19 screening.

Design, setting and participants

This prospective observational cohort included adults presenting with cough and/or dyspnea at a SARS-CoV-2 screening center of Lausanne University Hospital between March 31st and May 8th, 2020.

Interventions

Investigators recorded standardized LUS images and videos in 10 lung zones per subject. Two blinded independent experts reviewed LUS recording and classified abnormal findings according to pre-specified criteria to investigate their predictive value to diagnose SARS-CoV-2 infection according to PCR on nasopharyngeal swabs (COVID^{pos} vs COVID^{neg}).

Primary and secondary outcome measures

We finally combined LUS and clinical findings to derive a multivariate logistic regression diagnostic score.

Results

Of 134 included patients, 23% (n=30/134) were COVID^{pos} and 77% (n=103/134) were COVID^{neg}; 85%, (n=114/134) cases were previously healthy healthcare workers presenting within 2 to 5 days of symptom onset (IQR). Abnormal LUS findings were significantly more frequent in COVID^{pos} compared to COVID^{neg} (45% versus 26%, p=0.045) and mostly consisted of focal pathologic B-lines. Combining clinical findings in a multivariate logistic regression score had an area under the receiver-operating curve of 80.3% to detect COVID-19, and slightly improved to 84.5% with the addition of addition of LUS features.

For peer review only

Conclusions

COVID^{pos} patients are significantly more likely to have lung pathology by LUS. However, LUS has a insufficient sensitivity and is not an appropriate screening tool in outpatients. LUS only adds little value to clinical features alone.

Strengths and limitations of this study

- This is the first study assessing the diagnostic performance of LUS for COVID-19 in outpatients with mild acute respiratory tract infection. Acquisition and interpretation of LUS images and videos were standardized.
- Ultrasound experts interpreted all LUS image and videos.
- The study population consisted mainly of young and healthy healthcare workers , which prevents extrapolation of our results to an older and comorbid population.

For peer review only

Introduction

A year into the pandemic, Coronavirus Disease (COVID-19) remains a constant threat, overburdening the healthcare system. Current molecular diagnostic tests such as PCR and rapid antigen/antibody tests rely on consumables, which are vulnerable to shortages and saturation during exponential demand. The use of lung imaging as a diagnostic tool for COVID-19 has shown promises. Chest CT has a good sensitivity for patients triaged in emergency departments [1,2] and has even been able to detect pathology in asymptomatic cases, suggesting its potential as an early screening test in specific populations [3–5]. However, CT and even X-rays expose patients to ionizing radiation, are costly, and often not available in decentralized screening sites. Lung ultrasonography (LUS) is an alternative, consumable-free, easy-to-use, portable, non-radiating and non-invasive screening tool that can be performed at the bedside, with simple disinfection between patients and only a negligible cost of ultrasound gel as a consumable. It would allow immediate identification of infected patients at the point-of-care and be invaluable to the sustainable control of the pandemic. Its diagnostic performance for pneumonia has been established using chest CT as a gold standard [6]. For COVID-19, recent studies conducted in emergency departments showed several LUS patterns ranging from mild interstitial infiltrate, to lung consolidation, which correlated with disease progression and outcome [7,8]. However, these studies included mostly severe patients in emergency departments or intensive care units, which may lead to overoptimistic diagnostic performance of LUS due to a spectrum effect [9]. To our knowledge, no studies have described LUS findings in subjects with mild COVID-19.

This study aims to compare LUS characteristics between SARS-CoV-2 PCR-confirmed (COVID^{pos}) and PCR-negative (COVID^{neg}) patients in a screening center and explore LUS performance for identification of COVID-19 outpatients.

For peer review only

Methods

Study design, setting and population

This prospective cohort study recruited consecutive outpatients at the COVID-19 screening center in Lausanne University Hospital, Switzerland (CHUV) between March 31st and May 8th 2020. All adults (age ≥ 18 years) presenting at the center with cough and/or dyspnea and who fulfilled eligibility criteria for nasopharyngeal SARS-CoV-2 real time (Rt-) PCR according to the State recommendations at the time of the study were eligible. These State criteria were the presence of symptoms suggestive of COVID in a health worker or a subject with at least one vulnerability criterion, *i.e.* age ≥ 65 years old or having at least one comorbidity (obesity, diabetes, active cancer, chronic cardiovascular, pulmonary, liver, renal or inflammatory disease). Exclusion criteria were uninterpretable Rt-PCR results or absence of LUS recording. Written informed consent was obtained from all participants.

To ensure that LUS abnormal findings would be specific of a respiratory tract infection, we included a control group of healthy volunteers, matched for age (± 5 years), sex, and smoking status with $COVID^{pos}$ patients (Supplementary Table 1). These volunteers were asymptomatic during the previous 15 days (absence of odynophagia, cough, dyspnea, runny nose, fever, loss of smell or taste) and did not have a documented SARS-CoV-2 infection.

At inclusion, demographics, comorbidities, symptoms (including duration), and vital signs were collected using a standardized electronic case report form in REDCap® (Research Electronic Data Capture). Patients were subsequently classified as either $COVID^{pos}$ or $COVID^{neg}$ according to the SARS-CoV-2 RT-PCR results (at inclusion or at any time during the 30-day follow-up if the test was repeated for the same clinical episode). We assessed 30-day outcome by phone using a standardized interview (persistence of symptoms, secondary medical consultation, hospital admission, death). The healthy controls were classified in a third group (healthy control group).

For peer review only

Research ethics approval

The study was approved by the Swiss Ethics Committee of the canton of Vaud (CER-VD 2019-02283).

Patient and public involvement

Subjects were not involved in the design or conduct of this study.

Sample size

The minimum sample size required for this study was 100 patients with a clinical suspicion of COVID. It was calculated using a COVID prevalence of 20% and an estimated sensitivity of LUS to identify COVID^{pos} at 80% This sample size guarantees a power of 80% with a false discovery rate of 5% [10].

Lung ultrasonography

Three medical students performed image acquisitions in the triage site. They were trained in LUS images acquisition with a 1-hour e-learning course and a 1-hour face-to-face practical course with an expert radiologist (JYM). The first 10 acquisitions were done under direct supervision of an experienced board-certified expert (OP) who verified the quality of recorded images. Acquisition was standardized according to the "10-zone method" [11,12], consisting of five zones per hemithorax. Two images (sagittal and transverse) and 5 second videos were systematically recorded in every zone with a Butterfly IQ™ personal US system (Butterfly, Guiford, CT, USA), using the lung preset. The LUS probe and the electronic tablet were disinfected with an alcohol-based solution between each patient to avoid nosocomial spread [13].

For interpretation of LUS pathology, a physician experienced in LUS (TB) and an expert radiologist (JYM), blinded to patients' diagnoses, independently filled a standardized report form as previously described [8]. The following patterns were reported for every zone: (1) normal appearance (A lines, < 3 B lines), (2) pathologic B lines (≥3 B lines), (3) confluent B

For peer review only

lines, (4) thickening of the pleura with pleural line irregularities (subpleural consolidation < 1 cm) or (5) consolidation (≥ 1 cm). The presence of pleural effusion was also recorded. Discordance between the two readers were adjudicated by a third expert (OP). The abnormal images were summed up in a LUS score for each patient, as previously described [8,14,15].

Statistical analyses

Differences between $COVID^{pos}$ and $COVID^{neg}$ patients for all collected demographic and clinical features as well as LUS findings and LUS score were evaluated by Mann Whitney or chi-squared test, as appropriate. A bilateral p value <0.05 was considered as indicative of statistical significance. A multivariate logistic regression was built from 22, 15, 10 and 8 features using recursive feature elimination (RFE), originally including the following:

1) **LUS findings (n=10)**
- Number of pathological zones for each of the five patterns (normal, pathological B lines, confluent B lines, pleural thickening, consolidation) (n=5)
- A dichotomized variable for the presence/absence of the above four pathological patterns detected (n=4)
- Binary variables for the presence of multifocal disease (n=1)

2) **Symptoms at presentation (n=8)**
- Binary variables for the presence of cough, sputum, dyspnea, fever, anosmia, rhinorrhea, myalgia, and diarrhea

3) **Vital signs (n=3)**
- Continuous variables for temperature, oxygen saturation, and respiratory rate

4) **Epidemiological history (n=1)**
- Binary variable for a history of known unprotected contact with a COVID-19 case

For peer review only

Feature coefficients are presented, as well as their importance in ranked order from RFE . Performance at several stages of the RFE are reported, using the top 22, 15, 10 and 8 features. Models using just LUS or just clinical findings were also built.

Diagnostic performance is reported as sensitivity, specificity, positive and negative predictive values (PPV, NPV), positive and negative likelihood ratios (LR+, LR-) and area under the receiver-operator curve (AUC). Due to the dataset size, we report findings on the entire dataset.

A diagnostic score was derived from the summed coefficients, normalized within a range from -6 (COVID^{pos} highly unlikely) to +4 (COVID^{pos} highly likely) and the number of patients in each class are presented for each value of the score. The optimal cut-point was chosen using Youden index [16].

The kappa coefficient was calculated to measure the inter-rater agreement between the two LUS readers. R Core Team (2019) statistical software and python 3.0 with the sklearn library was used for analyses. Similar analyses were attempted on the outcome at 30-day follow up but impossible due to the limited sample size.

The reporting of our results followed the STARD guidelines.

For peer review only

Results

Demographics and clinical presentation

A total of 141 patients met inclusion criteria and were enrolled into the study; seven (5%) were later excluded, due to uninterpretable PCR results or LUS technical issues (hospital's network connection problems). Of the 134 remaining patients, 31 (23%) were classified as COVID^{pos} and 103 (77%) as COVID^{neg} based on Rt-PCR test. Among the 13 COVID^{neg} patients who had a second screening test during the 30-day follow-up, only one had a positive SARS-CoV-2 Rt-PCR, related to a clearly distinct clinical episode. This patient was thus classified as COVID^{neg}. Most patients were female (63%), healthcare workers (85%) with a median age of 35 years; most sought out testing within the first 5 days of symptom onset (Table 1). COVID^{pos} patients had fewer comorbidities than COVID^{neg}, the latter suffering mostly from asthma, obesity or hypertension. COVID^{pos} patients presented more often with a history of fever and anosmia, but less often with dyspnea than COVID^{neg} patients. Vital signs at inclusion were normal in most patients of both groups.

Lung ultrasonography findings

Lung ultrasound was abnormal in 31% of patients (Table 2). The two observers showed good concordance to differentiate a normal from an abnormal LUS, with a kappa of 0.67. Most anomalies were focal and unilateral. The most frequent patterns were pathologic B-lines and thickening of the pleura with pleural line irregularities. Only 9.1% of control subjects presented any abnormal finding on LUS, and all these anomalies were focal pathologic or confluent B lines (Supplementary Table 2).

Among all symptomatic patients, two factors were significantly associated with abnormal LUS: SARS-CoV-2 infection and history of fever (Table 3). Indeed, COVID^{pos} patients had abnormal LUS findings significantly more frequently compared with COVID^{neg} (45% versus 26%, p=0.045). However, this feature alone was poorly sensitive (45%) and specific (74%). No

For peer review only

specific ultrasonographic pattern on its own significantly distinguished $COVID^{pos}$ from $COVID^{neg}$ subjects (Table 2).

Although not statistically different, the proportion of COVID-19^{pos} with abnormal LUS findings was positively associated with symptoms duration. While only 30% of COVID-19^{pos} patients had abnormal LUS within 2 days of symptom onset, 52% of patients had pathological LUS after 2 days (p=0.24).

Multivariate diagnostic score.

We combined LUS findings with symptoms, vital signs and a binary feature for known contact with a COVID-19 case to build a multivariate logistic regression diagnostic score. Using all features, the score had 78.8% sensitivity, 84.0% specificity, 83.1% PPV, 61.4% NPV, 4.9 LR+, 0.3 LR- and 84.5% AUC (Figure 1). We present a plot on which to assess the score according to a desired sensitivity/specificity trade-off.

In Table 4, score performance with several combinations of features at various stages of RFE are presented. The strongest positive predictor was any evidence of pleural thickening at any number of sites (coefficient: +0.69) with LUS, although it became a negative predictor with an increasing number of sites with this feature (-0.40). The presence of pathological B lines and confluent pathological B lines were also positively associated with COVID infection in this score. All three of the above patterns were retained by RFE within the top seven features. The LUS features that were negative and quickly eliminated by RFE were those describing consolidation and multifocal pathology. Cough, fever and anosmia were the highest ranked symptoms (coefficient ≥0.4), in line with previous reports. While LUS patterns were highly ranked in the RFE, rerunning the model without LUS findings reduced AUC by only 4% (AUC 84.5% vs 80.3%). LUS findings were poorly sensitive in the absence of clinical features (AUC: 63.9% Sensitivity: 45.5%, Specificity: 77.3%, PPV: 66.7%, NPV: 55.6%, LR+: 2.0, LR-: 0.7).

For peer review only

224 Combining all 22 features and using RFE, we observe that removing 7 features had minimal impact on score performance, and removing 12 features reduces AUC by only 4% compared to the original.

30-day outcome

The 30-day follow-up was available for 121/134 (90%) patients. None was hospitalized or died during follow-up. COVID^{pos} patients had more frequently persistent symptoms (fatigue, dyspnea or anosmia) at 30-day compared with COVID^{neg} (Table 1).

The presence of an abnormal LUS at inclusion was not associated with symptom persistence (Table 3).

As no patients were admitted or died, we could not analyze the value of LUS findings to predict critical clinical outcome.

For peer review only

Discussion

Lung pathology is detectable by chest CT early in the course of COVID disease, even in asymptomatic patients, suggesting that lung imaging might have a place as a complementary diagnostic tool [3]. However, large scale CT screening is not feasible even in hospital settings with abundant resources. Point-of-care LUS is now affordable, portable and implementable in a decentralized setting and has all the attributes to become a pragmatic community-based screening tool.

We evaluated the diagnostic performance of LUS in a prospective cohort of subjects with mild acute respiratory tract infection attending a COVID-19 Swiss screening center. $COVID^{pos}$ outpatients more frequently had abnormal LUS findings at inclusion compared with $COVID^{neg}$. However, LUS findings alone had insufficient sensitivity, NPV and LR- to recommend LUS as an independent screening tool in outpatients. The combination of both LUS and clinical features in a multivariate regression score showed that LUS features only adds little value to clinical features alone regarding the prediction of COVID-19.

The limited sensitivity of LUS in our population is discordant with previous studies, which showed a good sensitivity (89-97%) to identify Rt-PCR-confirmed COVID-19. These retrospective studies were conducted in emergency departments and included patients with severe and critical COVID-19 infection[17–19]. Other studies using chest CT also showed an excellent sensitivity (97-98%) to diagnose COVID-19 [2,20,21]. However, all these studies were conducted in hospitalized patients with severe or critical disease, preventing extrapolation to our milder population screened for symptoms only.

The clinical severity of the disease strongly affects the performance of diagnostic tests, and particularly the sensitivity of LUS. We conclude that while LUS may be an interesting COVID-19 screening tool in emergency departments, it is not reliable when used alone in patients with mild disease. In the only study investigating chest CT features in patients with asymptomatic

For peer review only

(73%) or mild (27%) COVID-19, which was conducted in the passengers of the cruise ship *Diamond Princess*, 54% of asymptomatic patients and 79% of patients with mild disease presented opacities on chest CT. These results suggested the potential use of chest CT in clinical decision making [3]. Most opacities were located in the peripheral areas of the lung, where LUS is performant. Patients included in the *Diamond Princess* study were older compared with our study population (mean of 63 ± 15 years vs. 39 ± 13 years), a possible explanation for the lower proportion of patients with lung involvement in our study.

We observed more abnormal LUS findings in $COVID^{pos}$ patients who had more than 2 days of symptoms (52% versus 30%), although our results were not statistically significant. Concordant with our findings, a relationship between the duration of infection and the proportion of abnormal radiological findings has been described [22–24]. In one study, only 44% of patients presenting within 2 days of symptoms had an abnormal CT, while this proportion rose to 91% after 3 to 5 days and 96% after 5 days [24]. This study did not provide any data on COVID-19 severity. In another study using chest X-ray in patients admitted to the emergency department, the proportion of an abnormal chest X ray increased with the duration of symptoms (63% in the first 2 days to 84% after 9 days) [25].

In our study, most patients with abnormal LUS findings presented with focal pathologic B lines, confluent B lines or pleural thickening, irrespective of the etiology of the acute respiratory tract infection. Inclusion of healthy volunteers confirmed the causality between LUS findings and acute respiratory tract infections. Indeed, only 9% of healthy volunteers presented LUS anomalies (and all were focal pathologic B lines).

Two previous study showed that thickened pleural lines on LUS were significantly associated with COVID-19 [17,18]. However, in a third report, LUS findings were similar in both COVID-19 and non-COVID-19 patients [19].

Limitations

For peer review only

Our study has some limitations. First, most of our subjects were healthy and young healthcare workers, which prevents extrapolation of our results to an older and comorbid population. However, young, healthy subjects are of a prime importance in the management of the virus spread [26]. Second, SARS-CoV-2 Rt-PCR nasopharyngeal swab was used as the gold standard, and we might have missed some early infections when it has limited sensitivity [27]. However, it is considered as the reference diagnostic method. Furthermore, we sought to mitigate technical and sample collection error using validated nucleic acid amplification tests and a dedicated trained medical team performing nasopharyngeal swabs [28]. In addition, we had 30-day follow-up, which may have reduced the number of patients misclassified as $COVID^{neg}$. Third, medical students, and not ultrasound experts, performed LUS images and videos acquisition. However, they had a focused training by experts and followed a standardized image acquisition protocol. To better investigate the predictive potential of LUS findings, we built a multivariate score. The small sample size and high feature count (n= 22) exposes the model to the risk of overfitting. Thus, this score is not ready for clinical use, but rather is a mean to demonstrate the feature importance by RFE.

Conclusion

To our knowledge, this is the first study, which assessed the use of LUS in a screening center outpatient population with mild COVID-19. As disease severity plays an important role in the ultrasonographic findings, LUS is poorly sensitive as a SARS-CoV-2 screening tool in the context of mild community-level screening.

For peer review only

Declarations

Funding

This work was supported by an academic award of the Leenaards Foundation (to NBB), by the Foundation of Lausanne University Hospital, and the Emergency Department Lausanne University Hospital. The funding bodies had no role in the design of the study and collection, analysis and interpretation of data and in writing the manuscript.

Acknowledgements

We thank all the patients who accepted to participate and make this study possible. We thank all healthcare workers of the triage unit of the emergency department of the University Hospital of Lausanne, who supported the study and managed COVID-19 suspected patients.

For peer review only

Author contributions

JYM, OH, PC, NBB: study conception, study design, study performance, study management, data analysis, data interpretation and manuscript writing. SS, TB, JYM, OP: LUS images review, data interpretation and critical review of the manuscript.

TE, LB: LUS images recording, data interpretation and critical review of the manuscript.

MH, JC: data analysis, interpretation, visualisations and critical review of the manuscript.

All authors approved the final version of the manuscript and agreed to be accountable for all aspects of the work in ensuring that questions related to the accuracy or integrity of any part of the work are appropriately investigated and resolved.

NBB had full access to all the data in the study and takes responsibility for the integrity of the data and the accuracy of the data analysis.

Dataset available from https://zenodo.org/record/4617904#.Ya-gfi3pOu6

Conflicts of interest: none declared

References

1 Guan W, Ni Z, Hu Y, Liang W, Ou C, He J, *et al.* Clinical Characteristics of Coronavirus Disease 2019 in China. *N Engl J Med* 2020;**382**:1708–20.
2 Ai T, Yang Z, Hou H, Zhan C, Chen C, Lv W, *et al.* Correlation of Chest CT and RT-PCR Testing in Coronavirus Disease 2019 (COVID-19) in China: A Report of 1014 Cases. *Radiology* 2020;:200642.
3 Inui S, Fujikawa A, Jitsu M, Kunishima N, Watanabe S, Suzuki Y, *et al.* Chest CT Findings in Cases from the Cruise Ship “Diamond Princess” with Coronavirus Disease 2019 (COVID-19). *Radiol Cardiothorac Imaging* 2020;**2**:e200110.
4 Shi H, Han X, Jiang N, Cao Y, Alwalid O, Gu J, *et al.* Radiological findings from 81 patients with COVID-19 pneumonia in Wuhan, China: a descriptive study. *Lancet Infect Dis* 2020;**20**:425–34.
5 Islam N, Salameh J-P, Leeflang MM, Hooft L, McGrath TA, van der Pol CB, *et al.* Thoracic imaging tests for the diagnosis of COVID-19. *Cochrane Database Syst Rev* 2020;**11**:CD013639.
6 Orso D, Guglielmo N, Copetti R. Lung ultrasound in diagnosing pneumonia in the emergency department: a systematic review and meta-analysis. *Eur J Emerg Med* 2018;**25**:312–21.
7 Peng Q-Y, Wang X-T, Zhang L-N. Findings of lung ultrasonography of novel corona virus pneumonia during the 2019–2020 epidemic. *Intensive Care Med* 2020;:1–2.
8 Brahier T, Meuwly J-Y, Pantet O, Brochu Vez M-J, Gerhard Donnet H, Hartley M-A, *et al.* Lung ultrasonography for risk stratification in patients with COVID-19: a prospective observational cohort study. *Clin Infect Dis Off Publ Infect Dis Soc Am* Published Online First: 17 September 2020. doi:10.1093/cid/ciaa1408
9 Mulherin SA, Miller WC. Spectrum Bias or Spectrum Effect? Subgroup Variation in Diagnostic Test Evaluation. *Ann Intern Med* 2002;**137**:598.
10 Bujang MA, Adnan TH. Requirements for Minimum Sample Size for Sensitivity and Specificity Analysis. *J Clin Diagn Res JCDR* 2016;**10**:YE01–6.
11 Rambhia SH, D’Agostino CA, Noor A, Villani R, Naidich JJ, Pellerito JS. Thoracic Ultrasound: Technique, Applications, and Interpretation. *Curr Probl Diagn Radiol* 2017;**46**:305–16.
12 Volpicelli G, Elbarbary M, Blaivas M, Lichtenstein DA, Mathis G, Kirkpatrick AW, *et al.* International evidence-based recommendations for point-of-care lung ultrasound. *Intensive Care Med* 2012;**38**:577–91.
13 AIUM. Guidelines for Cleaning and Preparing External- and Internal-Use Ultrasound Transducers and Equipment Between Patients as well as Safe Handling and Use of Ultrasound Coupling Gel. 2020.
14 Mayo PH, Copetti R, Feller-Kopman D, Mathis G, Maury E, Mongodi S, *et al.* Thoracic ultrasonography: a narrative review. *Intensive Care Med* 2019;**45**:1200–11.
15 Soldati G, Smargiassi A, Inchingolo R, Buonsenso D, Perrone T, Briganti DF, *et al.* Proposal for International Standardization of the Use of Lung Ultrasound for Patients With COVID-19. *J Ultrasound Med*;**n/a**. doi:10.1002/jum.15285
16 Schisterman EF, Perkins NJ, Liu A, Bondell H. Optimal cut-point and its corresponding Youden Index to discriminate individuals using pooled blood samples. *Epidemiol Camb Mass* 2005;**16**:73–81.
17 Pare JR, Camelo I, Mayo KC, Leo MM, Dugas JN, Nelson KP, *et al.* Point-of-care Lung Ultrasound Is More Sensitive than Chest Radiograph for Evaluation of COVID-19. *West J Emerg Med* 2020;**21**:771–8.
18 Bar S, Lecourtois A, Diouf M, Goldberg E, Bourbon C, Arnaud E, *et al.* The

For peer review only

association of lung ultrasound images with COVID-19 infection in an emergency room cohort. *Anaesthesia* 2020;**75**:1620–5.
19 Sorlini C, Femia M, Nattino G, Bellone P, Gesu E, Francione P, *et al.* The role of lung ultrasound as a frontline diagnostic tool in the era of COVID-19 outbreak. *Intern Emerg Med* Published Online First: 22 October 2020. doi:10.1007/s11739-020-02524-8
20 Raptis CA, Hammer MM, Short RG, Shah A, Bhalla S, Bierhals AJ, *et al.* Chest CT and Coronavirus Disease (COVID-19): A Critical Review of the Literature to Date. *Am J Roentgenol* 2020;**215**:839–42.
21 Fang Y, Zhang H, Xie J, Lin M, Ying L, Pang P, *et al.* Sensitivity of Chest CT for COVID-19: Comparison to RT-PCR. *Radiology* 2020;**296**:E115–7.
22 Salehi S, Abedi A, Balakrishnan S, Gholamrezanezhad A. Coronavirus Disease 2019 (COVID-19): A Systematic Review of Imaging Findings in 919 Patients. *Am J Roentgenol* 2020;**215**:87–93.
23 Jin Y-H, Cai L, Cheng Z-S, Cheng H, Deng T, Fan Y-P, *et al.* A rapid advice guideline for the diagnosis and treatment of 2019 novel coronavirus (2019-nCoV) infected pneumonia (standard version). *Mil Med Res* 2020;**7**:4.
24 Bernheim A, Mei X, Huang M, Yang Y, Fayad ZA, Zhang N, *et al.* Chest CT Findings in Coronavirus Disease-19 (COVID-19): Relationship to Duration of Infection. *Radiology* 2020;**295**:200463.
25 Vancheri SG, Savietto G, Ballati F, Maggi A, Canino C, Bortolotto C, *et al.* Radiographic findings in 240 patients with COVID-19 pneumonia: time-dependence after the onset of symptoms. *Eur Radiol* 2020;**30**:6161–9.
26 Rivett L, Sridhar S, Sparkes D, Routledge M, Jones NK, Forrest S, *et al.* Screening of healthcare workers for SARS-CoV-2 highlights the role of asymptomatic carriage in COVID-19 transmission. *eLife* 2020;**9**. doi:10.7554/eLife.58728
27 Caruana G, Croxatto A, Coste AT, Opota O, Lamoth F, Jaton K, *et al.* Diagnostic strategies for SARS-CoV-2 infection and interpretation of microbiological results. *Clin Microbiol Infect* 2020;**26**:1178–82.
28 Opota O, Brouillet R, Greub G, Jaton K. Comparison of SARS-CoV-2 RT-PCR on a high-throughput molecular diagnostic platform and the cobas SARS-CoV-2 test for the diagnostic of COVID-19 on various clinical samples. *Pathog Dis* 2020;**78**:ftaa061.

For peer review only

Figure Legend

Figure 1. A multivariate logistic regression diagnostic score (x-axis) to discriminate COVID^{pos} from COVID^{neg} patients (black and white bars respectively with count on y axis). Sensitivity (—) and specificity (—) of the score are plotted with Youden's index (sensitivity + specificity -1) marked in orange. All 22 features are used in the depicted image on a model trained on all data points.

For peer review only

Tables

Table 1. Demographics, clinical characteristics and 30-day outcome of study participants according to nasopharyngeal Rt-PCR SARS-CoV-2 results

	All (n=134)	SARS-Co-V2 positive (n=31)	SARS-CoV-2 negative (n=103)	P value
**Demographics**				
Female sex	84 (63)	20 (65)	64 (62)	0.810
Age, years; Mean (SD)	35.5 [29, 46]	34 [26, 42]	37 [29, 50]	0.316
**Known contact with COVID subject**	33 (28)	10 (34)	23 (25)	0.334
Current smoker	39 (29)	7 (23)	32 (31)	0.362
Alcohol misuse	3 (2.2)	0 (0)	3 (2.9)	0.337
**Reason for testing**				
Vulnerable person[a]	20 (15)	6 (19)	14 (14)	0.430
Healthcare worker	114 (85)	25 (81)	89 (86)	0.430
**Comorbidities**				
Any	38 (28)	3 (9.7)	35 (34)	0.008
Hypertension	10 (7.5)	1 (3.2)	9 (8.7)	0.306
Diabetes	2 (1.)	0 (0)	2 (1.9)	0.434
Obesity	16 (12)	5 (16)	11 (11)	0.423
Asthma	17 (13)	1 (3.2)	16 (16)	0.071
Cardiovascular disease [b]	5 (3.7)	1 (3.2)	4 (3.9)	0.865
Pulmonary disease [c]	3 (2.2)	0 (0)	3 (2.9)	0.337
Active cancer	3 (2.2)	2 (6.5)	1 (1.0)	0.071
Hepatitis or liver cirrhosis	2 (1.4)	0 (0)	2 (1.9)	0.434
Chronic renal failure [d]	2 (1.4)	0 (0)	2 (1.9)	0.434
Chronic inflammatory disease	4 (3.0)	0 (0)	4 (3.9)	0.265
**Symptoms**				
Duration of symptoms, days; Median (IQR)	3 [2, 5]	3 [2, 4]	3 [2, 5]	0.942
Duration of symptoms				0.695
0-2 days	50 (38)	10 (32)	40 (39)	
3-5 days	57 (43)	18 (58)	39 (38)	

For peer review only

≥6 days	26 (20)	3 (9.7)	23 (23)	
Cough	118 (88)	30 (97)	88 (85)	0.088
Expectorations	27 (20)	10 (32)	17 (17)	0.055
Dyspnea	79 (59)	13 (42)	66 (64)	0.028
History of fever	75 (56)	23 (74)	52 (50)	0.020
Anosmia	24 (18)	10 (32)	14 (14)	0.017
Rhinorrhea	76 (57)	20 (65)	56 (54)	0.317
Odynophagia	55 (41)	13 (42)	42 (41)	0.908
Myalgia	91 (68)	25 (81)	66 (64)	0.083
Diarrhea	34 (25)	5 (16)	29 (28)	0.177
Temperature, °C; Median (IQR)	36.9 [36.6, 37.3]	37 [36.7, 37.5]	36.9 [36.6, 37.2]	0.202
Respiratory rate, beaths/minute; Median (IQR)	18 [16, 20]	18 [14, 20]	18 [16, 20]	0.236
Saturation, %; Median (IQR)	97 [97, 98]	98 [97, 98]	97 [97, 98]	0.403
Heart rate, beats/minute; Median (IQR)	86 [77, 95]	87 [79, 90]	86 [76, 98]	0.955
**Follow up at 30 days**				
Persistence of any symptoms at day 30	28 (23)	12 (41)	16 (17)	0.008
Fatigue	14 (10)	9 (29)	5 (4.9)	0.000
Myalgia	6 (4.5)	3 (9.7)	3 (2.9)	0.110
Cough	10 (7.4)	3 (9.7)	7 (6.8)	0.592
Expectoration	2 (1.4)	1 (3.2)	1 (0.97)	0.364
Dyspnea	9 (6.7)	6 (19)	3 (2.9)	0.001
Fever	2 (1.4)	1 (3.2)	1 (0.97)	0.364
Anosmia	8 (6.0)	7 (23)	1 (0.97)	0.000
Rhinorrhea	1 (0.8)	1 (3.2)	0 (0)	0.067
Odynodysphagia	2 (1.4)	1 (3.2)	1 (0.97)	0.364
Diarrhea	2 (1.4)	1 (3.2)	1 (0.97)	0.364
Medical consultation during follow-up	32 (26)	9 (31)	23 (25)	0.521
Hospitalization / Death	0 (0)	0 (0)	0 (0)	

Data are presented as n (%) unless indicated.
Missing values: contact with infected people, 15; medical consultation at inclusion, 1; vital signs, 5; duration of symptoms, 1; obesity, 1.
Abbreviations: IQR, interquartile range.
[a] ≥ 65 years old or comorbidity (obesity, diabetes, active cancer, chronic cardiovascular, pulmonary, liver, renal or inflammatory disease)
[b] Arrythmia, coronary disease.
[c] Chronic obstructive pulmonary disease, fibrosis.
[d] Stage III–V according to CKD classification.

For peer review only

421 **Table 2.** Lung ultrasound characteristics of study participants according to nasopharyngeal Rt-PCR SARS-CoV-2 results

	All (n=134)	SARS-CoV-2 positive (n=31)	SARS-CoV-2 negative (n=103)	P value
Abnormal lung ultrasound (any abnormal finding)	41 (31)	14 (45)	27 (26)	0.045
Abnormal lung ultrasound, apart from focal B-lines	30 (22)	11 (35)	19 (18)	0.046
Multifocal	16 (12)	6 (19)	10 (9.7)	0.146
Bilateral	8 (6.0)	3 (9.7)	5 (4.9)	0.320
Number of pathologic zones; Median (IQR)	0 [0, 1]	0 [0, 1]	0 [0, 1]	0.044
Pathologic B-lines (≥ 3)	20 (15)	6 (19)	14 (14)	0.430
Confluent B-lines (White lung)	11 (8.2)	4 (13)	7 (6.8)	0.277
Pleural thickening	18 (13)	6 (19)	12 (12)	0.270
Consolidations (> 1cm)	1 (0.75)	0 (0)	1 (0.97)	0.582
Pleural effusion	1 (.75)	0 (0)	1 (.97)	0.000
LUS score; Median (IQR)	0 [0, 1]	0 [0, 3]	0 [0, 1]	0.044

422

423

For peer review only

Table 3. Demographics and clinical characteristics of study participants according to the presence of an abnormal lung ultrasound

	All (n=134)	Abnormal LUS (n=41)	Normal LUS (n=93)	P value
**Demographics**				
Female sex	84 (63)	28 (68)	56 (60)	0.373
Age; Median (IQR)	35.5 [29, 46]	38 [31, 48]	35 [28, 45]	0.574
Current cigarette smoker	39 (29)	12 (29)	27 (29)	0.978
Alcohol misuse	3 (2.2)	0 (0)	3 (3.2)	0.245
**Reason of testing**				
Vulnerable person	20 (15)	3 (7.3)	17 (18)	0.101
Healthcare worker	114 (85)	38 (93)	76 (82)	0.101
**Positive Rt-PCR result**	31 (23)	14 (34)	17 (18)	0.045
**Comorbidities**				
Any	38 (28)	13 (32)	25 (27)	0.568
Hypertension	10 (7.5)	3 (7.3)	7 (7.5)	0.966
Diabetes	2 (1.5)	1 (2.4)	1 (1.1)	0.549
Obesity	16 (12)	3 (7.3)	13 (14)	0.265
Asthma	17 (13)	7 (17)	10 (11)	0.311
Cardiovascular disease [b]	5 (3.7)	2 (4.9)	3 (3.2)	0.642
Pulmonary disease [c]	3 (2.2)	0 (0)	3 (3.2)	0.245
Active cancer	3 (2.2)	1 (2.4)	2 (2.2)	0.917
Hepatitis or liver cirrhosis	2 (1.5)	1 (2.4)	1 (1.1)	0.549
Chronic renal failure [d]	2 (1.5)	0 (0)	2 (2.2)	0.344
Chronic inflammatory disease	4 (3.0)	0 (0)	4 (4.3)	0.178
**Symptoms**				
Duration of symptoms, days; Median (IQR)	3 [2, 5]	3 [2, 5]	3 [2, 5]	0.344
Duration of symptoms				0.210
0-2 days	50 (38)	11 (22)	39 (78)	
3-5 days	57 (43)	21 (37)	36 (63)	
≥ 6 days	26 (20)	9 (35)	17 (65)	
Cough	118 (88)	34 (83)	84 (90)	0.224

For peer review only

Expectorations	27 (20)	7 (17)	20 (22)	0.556
Dyspnea	79 (59)	25 (61)	54 (58)	0.752
Hemoptysis	2 (1.5)	0 (0)	2 (2.2)	0.344
History of fever	75 (56)	29 (71)	46 (49)	0.022
Anosmia	24 (18)	11 (27)	13 (14)	0.074
Rhinorrhea	76 (57)	21 (51)	55 (59)	0.394
Odynophagia	55 (41)	17 (41)	38 (41)	0.948
Myalgia	91 (68)	31 (76)	60 (65)	0.205
Diarrhea	34 (25)	8 (20)	26 (28)	0.301
Temperature, °C; Median (IQR)	36.9 [36.6, 37.3]	37 [36.6, 37.5]	36.9 [36.6, 37.2]	0.270
Respiratory rate, beaths/minute; Median (IQR)	18 [16, 20]	18 [16, 20]	18 [16, 20]	0.330
Saturation, %; Median (IQR)	97 [97, 98]	97 [97, 98]	97 [97, 98]	0.385
Heart rate, beats/minute; Median (IQR)	86 [77, 95]	88 [79, 98]	85 [76.5, 94]	0.170
**Follow-up at 30 days**				
Persistence of any symptoms at day 30	28 (23)	9 (24)	19 (23)	0.924
Fatigue	14 (10)	7 (17)	7 (7.5)	0.096
Myalgia	6 (4.5)	2 (4.9)	4 (4.3)	0.882
Cough	10 (7.5)	3 (7.3)	7 (7.5)	0.966
Expectorations	2 (1.5)	0 (0)	2 (2.2)	0.344
Dyspnea	9 (6.7)	4 (9.8)	5 (5.4)	0.351
Fever	2 (1.5)	0 (0)	2 (2.2)	0.344
Anosmia	8 (6.0)	1 (2.4)	7 (7.5)	0.252
Rhinorrhea	1 (.75)	0 (0)	1 (1.1)	0.505
Odynophagia	2 (1.5)	1 (2.4)	1 (1.1)	0.549
Diarrhea	2 (1.5)	0 (0)	2 (2.2)	0.344
Medical consultation during follow-up	26 (21)	10 (26)	16 (19)	0.364
Hospitalization/Death	0 (0)	0 (0)	0 (0)	

Data are presented as n (%) unless otherwise indicated.
Abbreviations: IQR, interquartile range.
[a] ≥ 65 years old or comorbidity (obesity, diabetes, active cancer, chronic cardiovascular, pulmonary, liver, renal or inflammatory disease)
[b] Arrythmia, coronary disease.
[c] Chronic obstructive pulmonary disease, fibrosis.
[d] Stage III–V according to CKD classification

For peer review only

Table 4. Multivariate logistic regression for COVID diagnosis

RFE selection order	Feature groups: LUS findings *(n=10)*	Feature groups: Symptoms *(n=8)*	Feature groups: Vital signs *(n=3)*	Feature groups: Epidemiological history *(n=1)*	Coefficient*: Neg	Coefficient*: Pos
**1** *(removed last)*		Cough				0.40
**2**	Pleural thickening (any)					0.69
**3**	Pleural thickening (number of sites)				-0.40	
**4**		Fever				0.44
**5**	Confluent B lines (number of sites)					0.41
**6**	Normal pattern (number of sites)					0.29
**7**	Pathologic B lines (number of sites)					0.49
**8**		Anosmia				0.43
**9**				Contact with COVID-19		0.47
**10**		Dyspnea			-0.28	
**11**		Myalgia				0.37
**12**		Diarrhea			-0.49	
13	Multifocality				-0.26	
**14**		Rhinorrhea				0.35
**15**		Sputum				0.41
**16**			Oxygen saturation			0.20
**17**	Consolidation (any)				-0.18	
**18**			Temperature (°C)			0.22

Diagnostic performance with various feature sets:

	**22-0 features=22** *10 LUS, 8 symptoms, 1 contact, 3 signs*	**22-7 features=15** *6 LUS, 8 symptoms, 1 contact, **NO** signs*	**22-12 features=10** *5 LUS, 4 symptoms, 1 contact, **NO** signs*	**22-14 features=8** *5 LUS, 3 symptoms, **NO** contact, **NO** signs*
	Sens: 78.8%	Sens: 75.8%	Sens: 84.8%	Sens: 81.8%
	Spec: 84.0%	Spec:83.2%	Spec: 72.3%	Spec: 62.2%
	AUC: 84.5%	AUC: 83.5%	AUC: 80.2%	AUC: 76.6%
	LR+: 4.9	LR+: 4.5	LR+: 3.1	LR+: 2.2
	LR-: 0.3	LR-: 0.3	LR-: 0.2	LR-: 0.3
	PPV: 83.1%	PPV: 81.8%	PPV: 75.4%	PPV: 68.4%
	NPV: 61.4%	NPV: 80.6%	NPV: 73.5%	PPV: 64.7%

**LUS findings only**	**Clinical only**
Sens: 45.5%	Sens: 72.7%
Spec: 77.3%	Spec: 79.8%
AUC: 63.9%	AUC: 80.3%

**19**			Respiratory rate		-0.30			LR+: 2.0	LR+: 3.6
**20**	Consolidation (any)				-0.18			LR-: 0.7	LR-: 0.3
**21**	Pathologic B lines (any)				-0.07			PPV: 66.7%	PPV: 78.3%
**22** *(removed first)*	Confluent B lines (any)				0.26			NPV: 55.6%	NPV: 64.5%

Multivariate logistic regression for COVID diagnosis where selection order is indirectly proportional to the feature's predictive importance, in recursive feature elimination (RFE), i.e., the feature labeled 22 was removed first, while 1 was retained until the end. Four feature groups containing 10 LUS findings, 8 symptoms, 3 vital signs and 1 epidemiological history of contact are color-coded according to their coefficient in the multivariate score including all 22 features (orange positive correlation with COVID and blue negative correlation). *The coefficient in multivariate scores is susceptible to multicollinearity.

For peer review only

438 **List of Supplemental Digital Content**

439 SupplementaryTables.docx

For peer review only

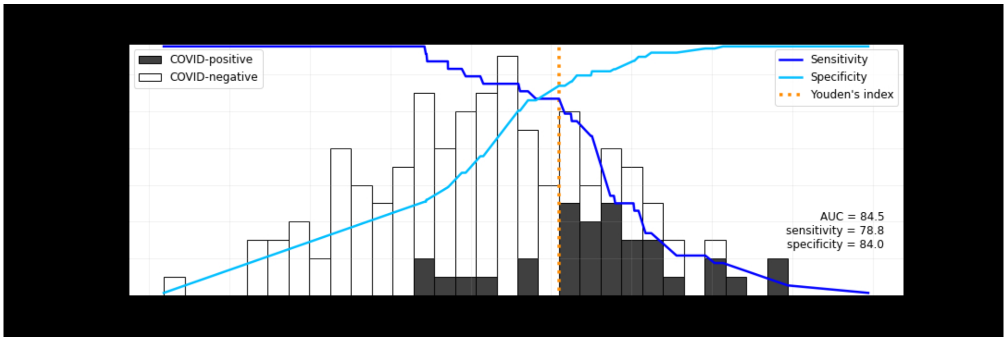

Figure 1. A multivariate logistic regression diagnostic score (x-axis) to discriminate COVIDpos from COVIDneg patients (black and white bars respectively with count on y axis). Sensitivity (—) and specificity (—) of the score are plotted with Youden's index (sensitivity + specificity -1) marked in orange. All 22 features are used in the depicted image on a model trained on all data points.

381x127mm (72 x 72 DPI)

Supplementary Tables.

Supplementary Table 1. Characteristics of study participants comparing healthy controls and patients with a lower respiratory tract infection ($COVID^{pos}$ and $COVID^{neg}$).

	All (n=178)	LRTI patients (n=134)	Control patients (n=44)	P value
Female sex	112 (63)	84 (63)	28 (64)	0.910
Age, years; Median (IQR)	34 [28, 45]	35 [29, 46]	31 [25, 42] *	0.007
Pulmonary disease [a]	3 (1.7)	3 (2.2)	0 (0)	0.317
Current cigarettes smoker	51 (29)	39 (29)	12 (27)	0.816

* $p < 0.05$
Data are presented as n (%) unless otherwise indicated.
Missing values: 0
Abbreviations: IQR, interquartile range; LRTI, Lower respiratory tract infection
[a] COPD, fibrosis.

For peer review only

Supplementary Table 2. Lung ultrasound characteristics of study participants comparing healthy controls and patients with a lower respiratory tract infection (COVID^{pos} and COVID^{neg}).

	**All (n=178)**	**LRTI patients (n=134)**	**Control patients (n=44)**	**P value**
Abnormal lung ultrasound	45 (25)	41 (31)	4 (9.1) *	0.004
Abnormal lung ultrasound apart from focal B lines	31 (17)	30 (22)	1 (2.2)	0.002
Multifocal	16 (9.0)	16 (12)	0 (0) *	0.016
Bilateral	8 (4.5)	8 (6.0)	0 (0)	0.097
Number of pathologic zones; Median (IQR)	0 [0, 0.7]	0 [0, 1]	0 [0, 0] *	0.003
Pathologic B lines (≥3)	23 (13)	20 (15)	3 (6.8)	0.164
Confluent B lines (White lung)	12 (6.7)	11 (8.2)	1 (2.3)	0.173
Thickening of the pleura with pleural line irregularities	18 (10)	18 (13)	0 (0) *	0.010
Consolidations (>1cm)	1 (0.6)	1 (0.8)	0 (0)	0.566
Pleural effusion	0 (0)	0 (0)	0 (0)	
LUS score; Median (IQR)	0 [0, 0.75]	0 [0, 1]	0 [0, 0] *	0.003

Data are presented as n (%) unless otherwise indicated.
Abbreviations: IQR, interquartile range.

For peer review only

STROBE Statement—Checklist of items that should be included in reports of ***cohort studies***

	Item No	Recommendation	Page No
**Title and abstract**	1	(*a*) Indicate the study's design with a commonly used term in the title or the abstract	1
		(*b*) Provide in the abstract an informative and balanced summary of what was done and what was found	2
**Introduction**			
Background/rationale	2	Explain the scientific background and rationale for the investigation being reported	4
Objectives	3	State specific objectives, including any prespecified hypotheses	4
**Methods**			
Study design	4	Present key elements of study design early in the paper	5
Setting	5	Describe the setting, locations, and relevant dates, including periods of recruitment, exposure, follow-up, and data collection	5
Participants	6	(*a*) Give the eligibility criteria, and the sources and methods of selection of participants. Describe methods of follow-up	5
		(*b*) For matched studies, give matching criteria and number of exposed and unexposed	
Variables	7	Clearly define all outcomes, exposures, predictors, potential confounders, and effect modifiers. Give diagnostic criteria, if applicable	7
Data sources/ measurement	8*	For each variable of interest, give sources of data and details of methods of assessment (measurement). Describe comparability of assessment methods if there is more than one group	8
Bias	9	Describe any efforts to address potential sources of bias	5
Study size	10	Explain how the study size was arrived at	6
Quantitative variables	11	Explain how quantitative variables were handled in the analyses. If applicable, describe which groupings were chosen and why	7
Statistical methods	12	(*a*) Describe all statistical methods, including those used to control for confounding	9
		(*b*) Describe any methods used to examine subgroups and interactions	7
		(*c*) Explain how missing data were addressed	9
		(*d*) If applicable, explain how loss to follow-up was addressed	N/A
		(*e*) Describe any sensitivity analyses	8
**Results**			
Participants	13*	(a) Report numbers of individuals at each stage of study—eg numbers potentially eligible, examined for eligibility, confirmed eligible, included in the study, completing follow-up, and analysed	9
		(b) Give reasons for non-participation at each stage	9
		(c) Consider use of a flow diagram	N/A
Descriptive data	14*	(a) Give characteristics of study participants (eg demographic, clinical, social) and information on exposures and potential confounders	9
		(b) Indicate number of participants with missing data for each variable of interest	21
		(c) Summarise follow-up time (eg, average and total amount)	11
Outcome data	15*	Report numbers of outcome events or summary measures over time	11

For peer review only

For peer review only - http://bmjopen.bmj.com/site/about/guidelines.xhtml

Main results	16	(*a*) Give unadjusted estimates and, if applicable, confounder-adjusted estimates and their precision (eg, 95% confidence interval). Make clear which confounders were adjusted for and why they were included<br>(*b*) Report category boundaries when continuous variables were categorized<br>(*c*) If relevant, consider translating estimates of relative risk into absolute risk for a meaningful time period	9
Other analyses	17	Report other analyses done—eg analyses of subgroups and interactions, and sensitivity analyses	10
**Discussion**			
Key results	18	Summarise key results with reference to study objectives	12
Limitations	19	Discuss limitations of the study, taking into account sources of potential bias or imprecision. Discuss both direction and magnitude of any potential bias	14
Interpretation	20	Give a cautious overall interpretation of results considering objectives, limitations, multiplicity of analyses, results from similar studies, and other relevant evidence	14
Generalisability	21	Discuss the generalisability (external validity) of the study results	14
**Other information**			
Funding	22	Give the source of funding and the role of the funders for the present study and, if applicable, for the original study on which the present article is based	15

*Give information separately for exposed and unexposed groups.

Note: An Explanation and Elaboration article discusses each checklist item and gives methodological background and published examples of transparent reporting. The STROBE checklist is best used in conjunction with this article (freely available on the Web sites of PLoS Medicine at http://www.plosmedicine.org/, Annals of Internal Medicine at http://www.annals.org/, and Epidemiology at http://www.epidem.com/). Information on the STROBE Initiative is available at http://www.strobe-statement.org.

For peer review only

For peer review only - http://bmjopen.bmj.com/site/about/guidelines.xhtml

BMJ Open

Point-of-care lung ultrasonography for early identification of mild COVID-19: a prospective cohort of outpatients in a Swiss screening center

Journal:	*BMJ Open*
Manuscript ID	bmjopen-2021-060181.R1
Article Type:	Original research
Date Submitted by the Author:	29-Mar-2022
Complete List of Authors:	Schaad, Siméon; Lausanne University Hospital<br>Brahier, Thomas; Lausanne University Hospital<br>HARTLEY, Mary-Anne; University of Lausanne, Digital global Health Department; EPFL<br>CORDONNIER, Jean-Baptiste; EPFL<br>BOSSO, Luca; Lausanne University Hospital Emergency Care Service, Emergency Department<br>ESPEJO, Tanguy; Lausanne University Hospital Emergency Care Service, Emergency Department<br>PANTET, Olivier; Lausanne University Hospital Adult Intensive Care Unit<br>Hugli, Olivier; University Hospital of Lausanne, Emergency department<br>Carron, Pierre-Nicolas; Centre Hospitalier Universitaire Vaudois, Emergency<br>MEUWLY, Jean-Yves; Lausanne University Hospital Division of Radio-diagnostics and Interventional Radiology, Department of Radiology<br>Boillat-Blanco, Noémie; Infectious Diseases Service
**Primary Subject Heading**:	Infectious diseases
Secondary Subject Heading:	Emergency medicine
Keywords:	COVID-19, ULTRASONOGRAPHY, Diagnostic radiology < RADIOLOGY & IMAGING, INFECTIOUS DISEASES

SCHOLARONE™
Manuscripts

I, the Submitting Author has the right to grant and does grant on behalf of all authors of the Work (as defined in the below author licence), an exclusive licence and/or a non-exclusive licence for contributions from authors who are: i) UK Crown employees; ii) where BMJ has agreed a CC-BY licence shall apply, and/or iii) in accordance with the terms applicable for US Federal Government officers or employees acting as part of their official duties; on a worldwide, perpetual, irrevocable, royalty-free basis to BMJ Publishing Group Ltd ("BMJ") its licensees and where the relevant Journal is co-owned by BMJ to the co-owners of the Journal, to publish the Work in this journal and any other BMJ products and to exploit all rights, as set out in our licence.

The Submitting Author accepts and understands that any supply made under these terms is made by BMJ to the Submitting Author unless you are acting as an employee on behalf of your employer or a postgraduate student of an affiliated institution which is paying any applicable article publishing charge ("APC") for Open Access articles. Where the Submitting Author wishes to make the Work available on an Open Access basis (and intends to pay the relevant APC), the terms of reuse of such Open Access shall be governed by a Creative Commons licence – details of these licences and which Creative Commons licence will apply to this Work are set out in our licence referred to above.

Other than as permitted in any relevant BMJ Author's Self Archiving Policies, I confirm this Work has not been accepted for publication elsewhere, is not being considered for publication elsewhere and does not duplicate material already published. I confirm all authors consent to publication of this Work and authorise the granting of this licence.

For peer review only

Point-of-care lung ultrasonography for early identification of mild COVID-19: a prospective cohort of outpatients in a Swiss screening center

Siméon SCHAAD[1*†], Thomas BRAHIER[1*], Mary-Anne HARTLEY[2,3], Jean-Baptiste CORDONNIER[3], Luca BOSSO[5], Tanguy ESPEJO[5], Olivier PANTET[4], Olivier HUGLI[5], Pierre-Nicolas CARRON[5], Jean-Yves MEUWLY[6*], Noémie BOILLAT-BLANCO[1*]

*Equal contribution to this work

†Corresponding author

Key words: COVID-19, lung ultrasound, screening, outpatients

[1] Infectious Diseases Service, University Hospital of Lausanne, Switzerland; [2] Digital global Health Department, Center for primary care and public health, University of Lausanne, Switzerland; [3] Machine Learning and Optimization Laboratory, EPFL, Switzerland; [4] Intensive Care Unit, University Hospital of Lausanne, Switzerland; [5] Emergency Department, University Hospital of Lausanne, Switzerland; [6] Department of Radiology, University Hospital of Lausanne, Switzerland.

Contact information: Siméon Schaad, Service of Infectious Diseases, University Hospital of Lausanne (CHUV), Rue du Bugnon 46, 1011 Lausanne, Switzerland, Phone: +41 79 524 15 85, E-mail: simeon.schaad@unil.ch

Word count : 3486

Funding and support: this work was supported by the Leenards foundation and by Lausanne University Hospital

For peer review only

Abstract

Objectives

Early identification of SARS-CoV-2 infection is important to guide quarantine and reduce transmission. This study evaluates the diagnostic performance of lung ultrasound (LUS), an affordable, consumable-free point-of-care tool, for COVID-19 screening.

Design, setting and participants

This prospective observational cohort included adults presenting with cough and/or dyspnea at a SARS-CoV-2 screening center of Lausanne University Hospital between March 31st and May 8th, 2020.

Interventions

Investigators recorded standardized LUS images and videos in 10 lung zones per subject. Two blinded independent experts reviewed LUS recording and classified abnormal findings according to pre-specified criteria to investigate their predictive value to diagnose SARS-CoV-2 infection according to PCR on nasopharyngeal swabs ($COVID^{pos}$ vs $COVID^{neg}$).

Primary and secondary outcome measures

We finally combined LUS and clinical findings to derive a multivariate logistic regression diagnostic score.

Results

Of 134 included patients, 23% (n=30/134) were $COVID^{pos}$ and 77% (n=103/134) were $COVID^{neg}$; 85%, (n=114/134) cases were previously healthy healthcare workers presenting within 2 to 5 days of symptom onset (IQR). Abnormal LUS findings were significantly more frequent in $COVID^{pos}$ compared to $COVID^{neg}$ (45% versus 26%, p=0.045) and mostly consisted of focal pathologic B-lines. Combining clinical findings in a multivariate logistic regression score had an area under the receiver-operating curve of 80.3% to detect COVID-19, and slightly improved to 84.5% with the addition of addition of LUS features.

For peer review only - http://bmjopen.bmj.com/site/about/guidelines.xhtml

Conclusions

COVID^{pos} patients are significantly more likely to have lung pathology by LUS. However, LUS has a insufficient sensitivity and is not an appropriate screening tool in outpatients. LUS only adds little value to clinical features alone.

Strengths and limitations of this study

- Acquisition and interpretation of LUS images and videos were standardized using predefined patterns.
- Ultrasound experts interpreted all LUS image and videos.
- The study population consisted mainly of young and healthy healthcare workers, which prevents extrapolation of our results to an older and comorbid population.

For peer review only

Introduction

A year into the pandemic, Coronavirus Disease (COVID-19) remains a constant threat, overburdening the healthcare system. Current molecular diagnostic tests such as PCR and rapid antigen/antibody tests rely on consumables, which are vulnerable to shortages and saturation during exponential demand. The use of lung imaging as a diagnostic tool for COVID-19 has shown promises. Chest CT has a good sensitivity for patients triaged in emergency departments [1,2] and has even been able to detect pathology in asymptomatic cases, suggesting its potential as an early screening test in specific populations [3–5]. However, CT and even X-rays expose patients to ionizing radiation, are costly, and often not available in decentralized screening sites. Lung ultrasonography (LUS) is an alternative, consumable-free, easy-to-use, portable, non-radiating and non-invasive screening tool that can be performed at the bedside, with simple disinfection between patients and only a negligible cost of ultrasound gel as a consumable. It would allow immediate identification of infected patients at the point-of-care and be invaluable to the sustainable control of the pandemic. Its diagnostic performance for pneumonia has been established using chest CT as a gold standard [6]. For COVID-19, recent studies conducted in emergency departments showed several LUS patterns ranging from mild interstitial infiltrate, to lung consolidation, which correlated with disease progression and outcome [7,8]. However, these studies included mostly severe patients in emergency departments or intensive care units, which may lead to overoptimistic diagnostic performance of LUS due to a spectrum effect [9]. To our knowledge, only one study included mild patients who did not need medical assessment, but the limited number of COVID positive patients prevents us from drawing a conclusion [10]. This study aims to compare LUS characteristics between SARS-CoV-2 PCR-confirmed ($COVID^{pos}$) and PCR-negative ($COVID^{neg}$) patients in a screening center and explore LUS performance for identification of COVID-19 outpatients.

For peer review only

Methods

Study design, setting and population

This prospective cohort study recruited consecutive outpatients at the COVID-19 screening center in Lausanne University Hospital, Switzerland (CHUV) between March 31st and May 8th 2020. All adults (age ≥ 18 years) presenting at the center with cough and/or dyspnea and who fulfilled eligibility criteria for nasopharyngeal SARS-CoV-2 real time (Rt-) PCR according to the State recommendations at the time of the study were eligible. These State criteria were the presence of symptoms suggestive of COVID in a health worker or a subject with at least one vulnerability criterion, *i.e.* age ≥ 65 years old or having at least one comorbidity (obesity, diabetes, active cancer, chronic cardiovascular, pulmonary, liver, renal or inflammatory disease). Exclusion criteria were uninterpretable Rt-PCR results or absence of LUS recording. Written informed consent was obtained from all participants.

To ensure that LUS abnormal findings would be specific of a respiratory tract infection, we included a control group of healthy volunteers, matched for age (± 5 years), sex, and smoking status with $COVID^{pos}$ patients (Supplementary Table 1). These volunteers were asymptomatic during the previous 15 days (absence of odynophagia, cough, dyspnea, runny nose, fever, loss of smell or taste) and did not have a documented SARS-CoV-2 infection.

At inclusion, demographics, comorbidities, symptoms (including duration), and vital signs were collected using a standardized electronic case report form in REDCap® (Research Electronic Data Capture). Patients were subsequently classified as either $COVID^{pos}$ or $COVID^{neg}$ according to the SARS-CoV-2 RT-PCR results (at inclusion or at any time during the 30-day follow-up if the test was repeated for the same clinical episode). We assessed 30-day outcome by phone using a standardized interview (persistence of symptoms, secondary medical consultation, hospital admission, death). The healthy controls were classified in a third group (healthy control group).

For peer review only - http://bmjopen.bmj.com/site/about/guidelines.xhtml

Research ethics approval

The study was approved by the Swiss Ethics Committee of the canton of Vaud (CER-VD 2019-02283).

Patient and public involvement

Subjects were not involved in the design or conduct of this study.

Sample size

The minimum sample size required for this study was 100 patients with a clinical suspicion of COVID. It was calculated using a COVID prevalence of 20% and an estimated sensitivity of LUS to identify $COVID^{pos}$ at 80% This sample size guarantees a power of 80% with a false discovery rate of 5% [11].

Lung ultrasonography

Three medical students performed image acquisitions in the triage site. They were trained in LUS images acquisition with a 1-hour e-learning course and a 1-hour face-to-face practical course with an expert radiologist (JYM). The first 10 acquisitions were done under direct supervision of an experienced board-certified expert (OP) who verified the quality of recorded images. Acquisition was standardized according to the “10-zone method” [12–14], consisting of five zones per hemithorax. Two images (sagittal and transverse) and 5 second videos were systematically recorded in every zone with a Butterfly IQ™ personal US system (Butterfly, Guiford, CT, USA), using the lung preset. The LUS probe and the electronic tablet were disinfected with an alcohol-based solution between each patient to avoid nosocomial spread [15].

For interpretation of LUS pathology, a physician experienced in LUS (TB) and an expert radiologist (JYM), blinded to patients’ diagnoses, independently filled a standardized report form as previously described [8]. The following patterns were reported for every zone: (1) normal appearance (A lines, < 3 B lines), (2) pathologic B lines (≥3 B lines), (3) confluent B

lines, (4) thickening of the pleura with pleural line irregularities (subpleural consolidation < 1 cm) or (5) consolidation (≥ 1 cm). The presence of pleural effusion was also recorded. Discordance between the two readers were adjudicated by a third expert (OP). The abnormal images were summed up in a LUS score for each patient, as previously described [8,16,17].

Statistical analyses

Differences between $COVID^{pos}$ and $COVID^{neg}$ patients for all collected demographic and clinical features as well as LUS findings and LUS score were evaluated by Mann Whitney or chi-squared test, as appropriate. A bilateral p value <0.05 was considered as indicative of statistical significance. A multivariate logistic regression was built from 22, 15, 10 and 8 features using recursive feature elimination (RFE), originally including the following:

1) LUS findings (n=10)

- Number of zones with each of the five patterns (normal, pathological B lines, confluent B lines, pleural thickening, consolidation) (n=5)
- A dichotomized variable for the presence/absence of the above four pathological patterns detected (n=4)
- Binary variables for the presence of multifocal disease (n=1)

2) Symptoms at presentation (n=8)

- Binary variables for the presence of cough, sputum, dyspnea, fever, anosmia, rhinorrhea, myalgia, and diarrhea

3) Vital signs (n=3)

- Continuous variables for temperature, oxygen saturation, and respiratory rate

4) Epidemiological history (n=1)

- Binary variable for a history of known unprotected contact with a COVID-19 case

For peer review only

Feature coefficients are presented, as well as their importance in ranked order from RFE. Performance at several stages of the RFE are reported, using the top 22, 15, 10 and 8 features. Models using just LUS or just clinical findings were also built.

Diagnostic performance is reported as sensitivity, specificity, positive and negative predictive values (PPV, NPV), positive and negative likelihood ratios (LR+, LR-) and area under the receiver-operator curve (AUC). Due to the dataset size, we report findings on the entire dataset.

A diagnostic score was derived from the summed coefficients, normalized within a range from -6 (COVID^{pos} highly unlikely) to +4 (COVID^{pos} highly likely) and the number of patients in each class are presented for each value of the score. The optimal cut-point was chosen using Youden index [18].

The kappa coefficient was calculated to measure the inter-rater agreement between the two LUS readers. R Core Team (2019) statistical software and python 3.0 with the sklearn library was used for analyses. Similar analyses were attempted on the outcome at 30-day follow up but impossible due to the limited sample size.

The reporting of our results followed the STARD guidelines.

For peer review only

Results

Demographics and clinical presentation

A total of 141 patients met inclusion criteria and were enrolled into the study; seven (5%) were later excluded, due to uninterpretable PCR results or LUS technical issues (hospital's network connection problems). Of the 134 remaining patients, 31 (23%) were classified as $COVID^{pos}$ and 103 (77%) as $COVID^{neg}$ based on Rt-PCR test. Among the 13 $COVID^{neg}$ patients who had a second screening test during the 30-day follow-up, only one had a positive SARS-CoV-2 Rt-PCR, related to a clearly distinct clinical episode. This patient was thus classified as $COVID^{neg}$. Most patients were female (63%), healthcare workers (85%) with a median age of 35 years; most sought out testing within the first 5 days of symptom onset (Table 1). $COVID^{pos}$ patients had fewer comorbidities than $COVID^{neg}$, the latter suffering mostly from asthma, obesity or hypertension. $COVID^{pos}$ patients presented more often with a history of fever and anosmia, but less often with dyspnea than $COVID^{neg}$ patients. Vital signs at inclusion were normal in most patients of both groups.

Lung ultrasonography findings

Lung ultrasound was abnormal in 31% of patients (Table 2). The two observers showed good concordance to differentiate a normal from an abnormal LUS, with a kappa of 0.67. Most anomalies were focal and unilateral. The most frequent patterns were pathologic B-lines and thickening of the pleura with pleural line irregularities. Only 9.1% of control subjects presented any abnormal finding on LUS, and all these anomalies were focal pathologic or confluent B lines (Supplementary Tables 2 and 3).

Among all symptomatic patients, two factors were significantly associated with abnormal LUS: SARS-CoV-2 infection and history of fever (Table 3). Indeed, $COVID^{pos}$ patients had abnormal LUS findings significantly more frequently compared with $COVID^{neg}$ (45% versus 26%, p=0.045). However, this feature alone was poorly sensitive (45%) and specific (74%). No

For peer review only

200 specific ultrasonographic pattern on its own significantly distinguished COVID^{pos} from
201 COVID^{neg} subjects (Table 2).

202 Although not statistically different, the proportion of COVID-19^{pos} with abnormal LUS
203 findings was positively associated with symptoms duration. While only 30% of COVID-19^{pos}
204 patients had abnormal LUS within 2 days of symptom onset, 52% of patients had pathological
205 LUS after 2 days (p=0.24).

206 ***Multivariate diagnostic score.***

207 We combined LUS findings with symptoms, vital signs and a binary feature for known contact
208 with a COVID-19 case to build a multivariate logistic regression diagnostic score. Using all
209 features, the score had 78.8% sensitivity, 84.0% specificity, 83.1% PPV, 61.4% NPV, 4.9 LR+,
210 0.3 LR- and 84.5% AUC (Figure 1). We present a plot on which to assess the score according
211 to a desired sensitivity/specificity trade-off.

212 In Table 4, score performance with several combinations of features at various stages of RFE
213 are presented. The strongest positive predictor was any evidence of pleural thickening at any
214 number of sites (coefficient: +0.69) with LUS, although it became a negative predictor with an
215 increasing number of sites with this feature (-0.40). The presence of pathological B lines and
216 confluent pathological B lines were also positively associated with COVID infection in this
217 score. All three of the above patterns were retained by RFE within the top seven features. The
218 LUS features that were negative and quickly eliminated by RFE were those describing
219 consolidation and multifocal pathology. Cough, fever and anosmia were the highest ranked
220 symptoms (coefficient ≥0.4), in line with previous reports. While LUS patterns were highly
221 ranked in the RFE, rerunning the model without LUS findings reduced AUC by only 4% (AUC
222 84.5% vs 80.3%). LUS findings were poorly sensitive in the absence of clinical features (AUC:
223 63.9% Sensitivity: 45.5%, Specificity: 77.3%, PPV: 66.7%, NPV: 55.6%, LR+: 2.0, LR-: 0.7).

For peer review only

Combining all 22 features and using RFE, we observe that removing 7 features had minimal impact on score performance, and removing 12 features reduces AUC by only 4% compared to the original.

30-day outcome

The 30-day follow-up was available for 121/134 (90%) patients. None was hospitalized or died during follow-up. $COVID^{pos}$ patients had more frequently persistent symptoms (fatigue, dyspnea or anosmia) at 30-day compared with $COVID^{neg}$ (Table 1).

The presence of an abnormal LUS at inclusion was not associated with symptom persistence (Table 3).

As no patients were admitted or died, we could not analyze the value of LUS findings to predict critical clinical outcome.

For peer review only

Discussion

Lung pathology is detectable by chest CT early in the course of COVID disease, even in asymptomatic patients, suggesting that lung imaging might have a place as a complementary diagnostic tool [3]. However, large scale CT screening is not feasible even in hospital settings with abundant resources. Point-of-care LUS is now affordable, portable and implementable in a decentralized setting and has all the attributes to become a pragmatic community-based screening tool.

We evaluated the diagnostic performance of LUS in a prospective cohort of subjects with mild acute respiratory tract infection attending a COVID-19 Swiss screening center. $COVID^{pos}$ outpatients more frequently had abnormal LUS findings at inclusion compared with $COVID^{neg}$. However, LUS findings alone had insufficient sensitivity, NPV and LR- to recommend LUS as an independent screening tool in outpatients. The combination of both LUS and clinical features in a multivariate regression score showed that LUS features only adds little value to clinical features alone regarding the prediction of COVID-19.

The limited sensitivity of LUS in our population is discordant with previous studies, which showed a sensitivity varying from 62 to 97% to identify Rt-PCR-confirmed COVID-19. These retrospective studies were conducted in emergency departments and included patients with severe and critical COVID-19 infection [19–21]. Some studies included mild patients who were evaluated in the ED and sometimes hospitalized[22–24]. Although these patients had mild COVID-19, their disease was more severe as they needed a medical assessment unlike the patients included in the present study who came for SARS-CoV2-screening.

Other studies using chest CT also showed an excellent sensitivity (97-98%) to diagnose COVID-19 [2,25,26]. However, all these studies were conducted in hospitalized patients, preventing extrapolation to our milder population screened for symptoms only.

For peer review only

The clinical severity of the disease strongly affects the performance of diagnostic tests, and particularly the sensitivity of LUS. We conclude that while LUS may be an interesting COVID-19 screening tool in emergency departments, it is not reliable when used alone in patients with mild disease. In the only study investigating chest CT features in patients with asymptomatic (73%) or mild (27%) COVID-19, which was conducted in the passengers of the cruise ship *Diamond Princess*, 54% of asymptomatic patients and 79% of patients with mild disease presented opacities on chest CT. These results suggested the potential use of chest CT in clinical decision making [3]. Most opacities were located in the peripheral areas of the lung, where LUS is performant. Patients included in the *Diamond Princess* study were older compared with our study population (mean of 63 ± 15 years vs. 39 ± 13 years), a possible explanation for the lower proportion of patients with lung involvement in our study.

Another potential explanation of the discrepancy between our study and previous publications is the short duration of symptoms at presentation. Although we did not confirm this association with our data, a previous study described a relationship between the duration of infection and the proportion of abnormal radiological findings[27–29]. In one study, only 44% of patients presenting within 2 days of symptoms had an abnormal CT, while this proportion rose to 91% after 3 to 5 days and 96% after 5 days [29]. This study did not provide any data on COVID-19 severity. In another study using chest X-ray in patients admitted to the emergency department, the proportion of an abnormal chest X ray increased with the duration of symptoms (63% in the first 2 days to 84% after 9 days) [30]. In our study, we did not confirm this hypothesis, however, we observed more abnormal LUS findings in $COVID^{pos}$ patients who had more than 2 days of symptoms (52% versus 30%), although our results were not statistically significant.

In our study, most patients with abnormal LUS findings presented with focal pathologic B lines, confluent B lines or pleural thickening, irrespective of the etiology of the acute respiratory tract infection. Inclusion of healthy volunteers confirmed the causality between LUS findings and

For peer review only

284 acute respiratory tract infections. Indeed, only 9% of healthy volunteers presented LUS anomalies (and all were focal pathologic B lines).

286 Two previous study showed that thickened pleural lines on LUS were significantly associated with COVID-19 [19,20]. However, in a third report, LUS findings were similar in both COVID-19 and non-COVID-19 patients [21].

289 **Limitations**

290 Our study has some limitations. First, most of our subjects were healthy and young healthcare workers, which prevents extrapolation of our results to an older and comorbid population. However, young, healthy subjects are of a prime importance in the management of the virus spread [31]. Second, SARS-CoV-2 Rt-PCR nasopharyngeal swab was used as the gold standard, and we might have missed some early infections when it has limited sensitivity [32]. However, it is considered as the reference diagnostic method. Furthermore, we sought to mitigate technical and sample collection error using validated nucleic acid amplification tests and a dedicated trained medical team performing nasopharyngeal swabs [33]. In addition, we had 30-day follow-up, which may have reduced the number of patients misclassified as $COVID^{neg}$. Third, medical students, and not ultrasound experts, performed LUS images and videos acquisition. However, they had a focused training by experts and followed a standardized image acquisition protocol. To better investigate the predictive potential of LUS findings, we built a multivariate score. The small sample size and high feature count (n= 22) exposes the model to the risk of overfitting. Thus, this score is not ready for clinical use, but rather is a mean to demonstrate the feature importance by RFE.

306 **Conclusion**

307 To our knowledge, this is the first study, which assessed the use of LUS in a screening center outpatient population with mild COVID-19. As disease severity plays an important role in the

For peer review only

ultrasonographic findings, LUS is poorly sensitive as a SARS-CoV-2 screening tool in the context of mild community-level screening.

For peer review only

Declarations

Funding

This work was supported by an academic award of the Leenaards Foundation (to NBB), by the Foundation of Lausanne University Hospital, and the Emergency Department Lausanne University Hospital. The funding bodies had no role in the design of the study and collection, analysis and interpretation of data and in writing the manuscript.

Acknowledgements

We thank all the patients who accepted to participate and make this study possible. We thank all healthcare workers of the triage unit of the emergency department of the University Hospital of Lausanne, who supported the study and managed COVID-19 suspected patients.

Author contributions

JYM, OH, PC, NBB: study conception, study design, study performance, study management, data analysis, data interpretation and manuscript writing. SS, TB, JYM, OP: LUS images review, data interpretation and critical review of the manuscript.

TE, LB: LUS images recording, data interpretation and critical review of the manuscript.

MH, JC: data analysis, interpretation, visualisations and critical review of the manuscript.

All authors approved the final version of the manuscript and agreed to be accountable for all aspects of the work in ensuring that questions related to the accuracy or integrity of any part of the work are appropriately investigated and resolved.

NBB had full access to all the data in the study and takes responsibility for the integrity of the data and the accuracy of the data analysis.

[dataset]Dataset available from Schaad et al. (2021). Point-of-care lung ultrasonography for early identification of mild COVID-19: a prospective cohort of outpatients in a Swiss screening center [Data set]. Zenodo. March 18, 2021 https://doi.org/10.5281/zenodo.4617904[34]

For peer review only

Conflicts of interest: none declared

For peer review only

References

1 Guan W, Ni Z, Hu Y, Liang W, Ou C, He J, *et al.* Clinical Characteristics of Coronavirus Disease 2019 in China. *N Engl J Med* 2020;**382**:1708–20.

2 Ai T, Yang Z, Hou H, Zhan C, Chen C, Lv W, *et al.* Correlation of Chest CT and RT-PCR Testing in Coronavirus Disease 2019 (COVID-19) in China: A Report of 1014 Cases. *Radiology* 2020;:200642.

3 Inui S, Fujikawa A, Jitsu M, Kunishima N, Watanabe S, Suzuki Y, *et al.* Chest CT Findings in Cases from the Cruise Ship “Diamond Princess” with Coronavirus Disease 2019 (COVID-19). *Radiol Cardiothorac Imaging* 2020;**2**:e200110.

4 Shi H, Han X, Jiang N, Cao Y, Alwalid O, Gu J, *et al.* Radiological findings from 81 patients with COVID-19 pneumonia in Wuhan, China: a descriptive study. *Lancet Infect Dis* 2020;**20**:425–34.

5 Islam N, Salameh J-P, Leeflang MM, Hooft L, McGrath TA, van der Pol CB, *et al.* Thoracic imaging tests for the diagnosis of COVID-19. *Cochrane Database Syst Rev* 2020;**11**:CD013639.

6 Orso D, Guglielmo N, Copetti R. Lung ultrasound in diagnosing pneumonia in the emergency department: a systematic review and meta-analysis. *Eur J Emerg Med* 2018;**25**:312–21.

7 Peng Q-Y, Wang X-T, Zhang L-N. Findings of lung ultrasonography of novel corona virus pneumonia during the 2019–2020 epidemic. *Intensive Care Med* 2020;:1–2.

8 Brahier T, Meuwly J-Y, Pantet O, Brochu Vez M-J, Gerhard Donnet H, Hartley M-A, *et al.* Lung ultrasonography for risk stratification in patients with COVID-19: a prospective observational cohort study. *Clin Infect Dis Off Publ Infect Dis Soc Am* Published Online First: 17 September 2020. doi:10.1093/cid/ciaa1408

9 Mulherin SA, Miller WC. Spectrum Bias or Spectrum Effect? Subgroup Variation in Diagnostic Test Evaluation. *Ann Intern Med* 2002;**137**:598.

10 Copetti R, Amore G, Giudice CA, Orso D, Cola S, Pillinini P, *et al.* Lung Ultrasonography in Ruling Out COVID-19 Among Health Care Workers in Two Italian Emergency Departments: A Multicenter Study. *J Diagn Med Sonogr* 2022;**38**:45–51.

11 Bujang MA, Adnan TH. Requirements for Minimum Sample Size for Sensitivity and Specificity Analysis. *J Clin Diagn Res JCDR* 2016;**10**:YE01–6.

12 Rambhia SH, D’Agostino CA, Noor A, Villani R, Naidich JJ, Pellerito JS. Thoracic Ultrasound: Technique, Applications, and Interpretation. *Curr Probl Diagn Radiol* 2017;**46**:305–16.

13 Volpicelli G, Elbarbary M, Blaivas M, Lichtenstein DA, Mathis G, Kirkpatrick AW, *et al.* International evidence-based recommendations for point-of-care lung ultrasound. *Intensive Care Med* 2012;**38**:577–91.

14 Russell FM, Ferre R, Ehrman RR, Noble V, Gargani L, Collins SP, *et al.* What are the minimum requirements to establish proficiency in lung ultrasound training for quantifying B-lines? *ESC Heart Fail* 2020;**7**:2941–7.

15 AIUM. Guidelines for Cleaning and Preparing External- and Internal-Use Ultrasound Transducers and Equipment Between Patients as well as Safe Handling and Use of Ultrasound Coupling Gel. 2020.

16 Mayo PH, Copetti R, Feller-Kopman D, Mathis G, Maury E, Mongodi S, *et al.* Thoracic ultrasonography: a narrative review. *Intensive Care Med* 2019;**45**:1200–11.

17 Soldati G, Smargiassi A, Inchingolo R, Buonsenso D, Perrone T, Briganti DF, *et al.* Proposal for International Standardization of the Use of Lung Ultrasound for Patients With COVID-19. *J Ultrasound Med*;**n/a**. doi:10.1002/jum.15285

18 Schisterman EF, Perkins NJ, Liu A, Bondell H. Optimal cut-point and its

For peer review only

corresponding Youden Index to discriminate individuals using pooled blood samples. *Epidemiol Camb Mass* 2005;**16**:73–81.

19 Pare JR, Camelo I, Mayo KC, Leo MM, Dugas JN, Nelson KP, *et al.* Point-of-care Lung Ultrasound Is More Sensitive than Chest Radiograph for Evaluation of COVID-19. *West J Emerg Med* 2020;**21**:771–8.

20 Bar S, Lecourtois A, Diouf M, Goldberg E, Bourbon C, Arnaud E, *et al.* The association of lung ultrasound images with COVID-19 infection in an emergency room cohort. *Anaesthesia* 2020;**75**:1620–5.

21 Sorlini C, Femia M, Nattino G, Bellone P, Gesu E, Francione P, *et al.* The role of lung ultrasound as a frontline diagnostic tool in the era of COVID-19 outbreak. *Intern Emerg Med* Published Online First: 22 October 2020. doi:10.1007/s11739-020-02524-8

22 Volpicelli G, Gargani L, Perlini S, Spinelli S, Barbieri G, Lanotte A, *et al.* Lung ultrasound for the early diagnosis of COVID-19 pneumonia: an international multicenter study. *Intensive Care Med* 2021;**47**:444–54.

23 Lichter Y, Topilsky Y, Taieb P, Banai A, Hochstadt A, Merdler I, *et al.* Lung ultrasound predicts clinical course and outcomes in COVID-19 patients. *Intensive Care Med* 2020;**46**:1873–83.

24 Speidel V, Conen A, Gisler V, Fux CA, Haubitz S. Lung Assessment with Point-of-Care Ultrasound in Respiratory Coronavirus Disease (COVID-19): A Prospective Cohort Study. *Ultrasound Med Biol* 2021;**47**:896–901.

25 Raptis CA, Hammer MM, Short RG, Shah A, Bhalla S, Bierhals AJ, *et al.* Chest CT and Coronavirus Disease (COVID-19): A Critical Review of the Literature to Date. *Am J Roentgenol* 2020;**215**:839–42.

26 Fang Y, Zhang H, Xie J, Lin M, Ying L, Pang P, *et al.* Sensitivity of Chest CT for COVID-19: Comparison to RT-PCR. *Radiology* 2020;**296**:E115–7.

27 Salehi S, Abedi A, Balakrishnan S, Gholamrezanezhad A. Coronavirus Disease 2019 (COVID-19): A Systematic Review of Imaging Findings in 919 Patients. *Am J Roentgenol* 2020;**215**:87–93.

28 Jin Y-H, Cai L, Cheng Z-S, Cheng H, Deng T, Fan Y-P, *et al.* A rapid advice guideline for the diagnosis and treatment of 2019 novel coronavirus (2019-nCoV) infected pneumonia (standard version). *Mil Med Res* 2020;**7**:4.

29 Bernheim A, Mei X, Huang M, Yang Y, Fayad ZA, Zhang N, *et al.* Chest CT Findings in Coronavirus Disease-19 (COVID-19): Relationship to Duration of Infection. *Radiology* 2020;**295**:200463.

30 Vancheri SG, Savietto G, Ballati F, Maggi A, Canino C, Bortolotto C, *et al.* Radiographic findings in 240 patients with COVID-19 pneumonia: time-dependence after the onset of symptoms. *Eur Radiol* 2020;**30**:6161–9.

31 Rivett L, Sridhar S, Sparkes D, Routledge M, Jones NK, Forrest S, *et al.* Screening of healthcare workers for SARS-CoV-2 highlights the role of asymptomatic carriage in COVID-19 transmission. *eLife* 2020;**9**. doi:10.7554/eLife.58728

32 Caruana G, Croxatto A, Coste AT, Opota O, Lamoth F, Jaton K, *et al.* Diagnostic strategies for SARS-CoV-2 infection and interpretation of microbiological results. *Clin Microbiol Infect* 2020;**26**:1178–82.

33 Opota O, Brouillet R, Greub G, Jaton K. Comparison of SARS-CoV-2 RT-PCR on a high-throughput molecular diagnostic platform and the cobas SARS-CoV-2 test for the diagnostic of COVID-19 on various clinical samples. *Pathog Dis* 2020;**78**:ftaa061.

[dataset]34 Schaad, Siméon, Brahier, Thomas, Hartley, Mary-Anne, Cordonnier, Jean-Baptiste, Bosso, Luca, Espejo, Tanguy, *et al.* Point-of-care lung ultrasonography for early identification of mild COVID-19: a prospective cohort of outpatients in a Swiss screening center. 2021. doi:10.5281/ZENODO.4617904

For peer review only

Figure Legend

Figure 1. A multivariate logistic regression diagnostic score (x-axis) to discriminate $COVID^{pos}$ from $COVID^{neg}$ patients (black and white bars respectively with count on y axis). Sensitivity (—) and specificity (—) of the score are plotted with Youden's index (sensitivity + specificity -1) marked in orange. All 22 features are used in the depicted image on a model trained on all data points.

For peer review only

Tables

Table 1. Demographics, clinical characteristics and 30-day outcome of study participants according to nasopharyngeal Rt-PCR SARS-CoV-2 results

	All (n=134)	SARS-Co-V2 positive (n=31)	SARS-CoV-2 negative (n=103)	P value
**Demographics**				
Female sex	84 (63)	20 (65)	64 (62)	0.810
Age, years; Mean (SD)	35.5 [29, 46]	34 [26, 42]	37 [29, 50]	0.316
**Known contact with COVID subject**	33 (28)	10 (34)	23 (25)	0.334
Current smoker	39 (29)	7 (23)	32 (31)	0.362
Alcohol misuse	3 (2.2)	0 (0)	3 (2.9)	0.337
**Reason for testing**				
Vulnerable person[a]	20 (15)	6 (19)	14 (14)	0.430
Healthcare worker	114 (85)	25 (81)	89 (86)	0.430
**Comorbidities**				
Any	38 (28)	3 (9.7)	35 (34)	0.008
Hypertension	10 (7.5)	1 (3.2)	9 (8.7)	0.306
Diabetes	2 (1.)	0 (0)	2 (1.9)	0.434
Obesity	16 (12)	5 (16)	11 (11)	0.423
Asthma	17 (13)	1 (3.2)	16 (16)	0.071
Cardiovascular disease [b]	5 (3.7)	1 (3.2)	4 (3.9)	0.865
Pulmonary disease [c]	3 (2.2)	0 (0)	3 (2.9)	0.337
Active cancer	3 (2.2)	2 (6.5)	1 (1.0)	0.071
Hepatitis or liver cirrhosis	2 (1.4)	0 (0)	2 (1.9)	0.434
Chronic renal failure [d]	2 (1.4)	0 (0)	2 (1.9)	0.434
Chronic inflammatory disease	4 (3.0)	0 (0)	4 (3.9)	0.265
**Symptoms**				
Duration of symptoms, days; Median (IQR)	3 [2, 5]	3 [2, 4]	3 [2, 5]	0.942
Duration of symptoms				0.695
0-2 days	50 (38)	10 (32)	40 (39)	
3-5 days	57 (43)	18 (58)	39 (38)	

For peer review only

≥6 days	26 (20)	3 (9.7)	23 (23)	
Cough	118 (88)	30 (97)	88 (85)	0.088
Expectorations	27 (20)	10 (32)	17 (17)	0.055
Dyspnea	79 (59)	13 (42)	66 (64)	0.028
History of fever	75 (56)	23 (74)	52 (50)	0.020
Anosmia	24 (18)	10 (32)	14 (14)	0.017
Rhinorrhea	76 (57)	20 (65)	56 (54)	0.317
Odynophagia	55 (41)	13 (42)	42 (41)	0.908
Myalgia	91 (68)	25 (81)	66 (64)	0.083
Diarrhea	34 (25)	5 (16)	29 (28)	0.177
Temperature, °C; Median (IQR)	36.9 [36.6, 37.3]	37 [36.7, 37.5]	36.9 [36.6, 37.2]	0.202
Respiratory rate, beaths/minute; Median (IQR)	18 [16, 20]	18 [14, 20]	18 [16, 20]	0.236
Saturation, %; Median (IQR)	97 [97, 98]	98 [97, 98]	97 [97, 98]	0.403
Heart rate, beats/minute; Median (IQR)	86 [77, 95]	87 [79, 90]	86 [76, 98]	0.955
**Follow up at 30 days**				
Persistence of any symptoms at day 30	28 (23)	12 (41)	16 (17)	0.008
Fatigue	14 (10)	9 (29)	5 (4.9)	0.000
Myalgia	6 (4.5)	3 (9.7)	3 (2.9)	0.110
Cough	10 (7.4)	3 (9.7)	7 (6.8)	0.592
Expectoration	2 (1.4)	1 (3.2)	1 (0.97)	0.364
Dyspnea	9 (6.7)	6 (19)	3 (2.9)	0.001
Fever	2 (1.4)	1 (3.2)	1 (0.97)	0.364
Anosmia	8 (6.0)	7 (23)	1 (0.97)	0.000
Rhinorrhea	1 (0.8)	1 (3.2)	0 (0)	0.067
Odynodysphagia	2 (1.4)	1 (3.2)	1 (0.97)	0.364
Diarrhea	2 (1.4)	1 (3.2)	1 (0.97)	0.364
Medical consultation during follow-up	32 (26)	9 (31)	23 (25)	0.521
Hospitalization / Death	0 (0)	0 (0)	0 (0)	

Data are presented as n (%) unless indicated.
Missing values: contact with infected people, 15; medical consultation at inclusion, 1; vital signs, 5; duration of symptoms, 1; obesity, 1.
Abbreviations: IQR, interquartile range.
[a] ≥ 65 years old or comorbidity (obesity, diabetes, active cancer, chronic cardiovascular, pulmonary, liver, renal or inflammatory disease)
[b] Arrythmia, coronary disease.
[c] Chronic obstructive pulmonary disease, fibrosis.
[d] Stage III–V according to CKD classification.

For peer review only

448 **Table 2.** Lung ultrasound characteristics of study participants according to nasopharyngeal Rt-PCR SARS-CoV-2 results

	**All (n=134)**	**SARS-CoV-2 positive (n=31)**	**SARS-CoV-2 negative (n=103)**	**P value**
Abnormal lung ultrasound (any abnormal finding)	41 (31)	14 (45)	27 (26)	0.045
Abnormal lung ultrasound, apart from focal B-lines	30 (22)	11 (35)	19 (18)	0.046
Multifocal	16 (12)	6 (19)	10 (9.7)	0.146
Bilateral	8 (6.0)	3 (9.7)	5 (4.9)	0.320
Number of pathologic zones; Median (IQR)	0 [0, 1]	0 [0, 1]	0 [0, 1]	0.044
Pathologic B-lines (≥ 3)	20 (15)	6 (19)	14 (14)	0.430
Confluent B-lines (White lung)	11 (8.2)	4 (13)	7 (6.8)	0.277
Pleural thickening	18 (13)	6 (19)	12 (12)	0.270
Consolidations (> 1cm)	1 (0.75)	0 (0)	1 (0.97)	0.582
Pleural effusion	1 (.75)	0 (0)	1 (.97)	0.000
LUS score; Median (IQR)	0 [0, 1]	0 [0, 3]	0 [0, 1]	0.044

449

450

For peer review only

451 **Table 3**. Demographics and clinical characteristics of study participants according to the presence of an abnormal lung ultrasound

	All (n=134)	Abnormal LUS (n=41)	Normal LUS (n=93)	P value
**Demographics**				
Female sex	84 (63)	28 (68)	56 (60)	0.373
Age; Median (IQR)	35.5 [29, 46]	38 [31, 48]	35 [28, 45]	0.574
Current cigarette smoker	39 (29)	12 (29)	27 (29)	0.978
Alcohol misuse	3 (2.2)	0 (0)	3 (3.2)	0.245
**Reason of testing**				
Vulnerable person	20 (15)	3 (7.3)	17 (18)	0.101
Healthcare worker	114 (85)	38 (93)	76 (82)	0.101
**Positive Rt-PCR result**	31 (23)	14 (34)	17 (18)	0.045
**Comorbidities**				
Any	38 (28)	13 (32)	25 (27)	0.568
Hypertension	10 (7.5)	3 (7.3)	7 (7.5)	0.966
Diabetes	2 (1.5)	1 (2.4)	1 (1.1)	0.549
Obesity	16 (12)	3 (7.3)	13 (14)	0.265
Asthma	17 (13)	7 (17)	10 (11)	0.311
Cardiovascular disease [b]	5 (3.7)	2 (4.9)	3 (3.2)	0.642
Pulmonary disease [c]	3 (2.2)	0 (0)	3 (3.2)	0.245
Active cancer	3 (2.2)	1 (2.4)	2 (2.2)	0.917
Hepatitis or liver cirrhosis	2 (1.5)	1 (2.4)	1 (1.1)	0.549
Chronic renal failure [d]	2 (1.5)	0 (0)	2 (2.2)	0.344
Chronic inflammatory disease	4 (3.0)	0 (0)	4 (4.3)	0.178
**Symptoms**				
Duration of symptoms, days; Median (IQR)	3 [2, 5]	3 [2, 5]	3 [2, 5]	0.344
Duration of symptoms				0.210
0-2 days	50 (38)	11 (22)	39 (78)	
3-5 days	57 (43)	21 (37)	36 (63)	
≥ 6 days	26 (20)	9 (35)	17 (65)	
Cough	118 (88)	34 (83)	84 (90)	0.224

For peer review only

Expectorations	27 (20)	7 (17)	20 (22)	0.556
Dyspnea	79 (59)	25 (61)	54 (58)	0.752
Hemoptysis	2 (1.5)	0 (0)	2 (2.2)	0.344
History of fever	75 (56)	29 (71)	46 (49)	0.022
Anosmia	24 (18)	11 (27)	13 (14)	0.074
Rhinorrhea	76 (57)	21 (51)	55 (59)	0.394
Odynophagia	55 (41)	17 (41)	38 (41)	0.948
Myalgia	91 (68)	31 (76)	60 (65)	0.205
Diarrhea	34 (25)	8 (20)	26 (28)	0.301
Temperature, °C; Median (IQR)	36.9 [36.6, 37.3]	37 [36.6, 37.5]	36.9 [36.6, 37.2]	0.270
Respiratory rate, beaths/minute; Median (IQR)	18 [16, 20]	18 [16, 20]	18 [16, 20]	0.330
Saturation, %; Median (IQR)	97 [97, 98]	97 [97, 98]	97 [97, 98]	0.385
Heart rate, beats/minute; Median (IQR)	86 [77, 95]	88 [79, 98]	85 [76.5, 94]	0.170
**Follow-up at 30 days**				
Persistence of any symptoms at day 30	28 (23)	9 (24)	19 (23)	0.924
Fatigue	14 (10)	7 (17)	7 (7.5)	0.096
Myalgia	6 (4.5)	2 (4.9)	4 (4.3)	0.882
Cough	10 (7.5)	3 (7.3)	7 (7.5)	0.966
Expectorations	2 (1.5)	0 (0)	2 (2.2)	0.344
Dyspnea	9 (6.7)	4 (9.8)	5 (5.4)	0.351
Fever	2 (1.5)	0 (0)	2 (2.2)	0.344
Anosmia	8 (6.0)	1 (2.4)	7 (7.5)	0.252
Rhinorrhea	1 (.75)	0 (0)	1 (1.1)	0.505
Odynophagia	2 (1.5)	1 (2.4)	1 (1.1)	0.549
Diarrhea	2 (1.5)	0 (0)	2 (2.2)	0.344
Medical consultation during follow-up	26 (21)	10 (26)	16 (19)	0.364
Hospitalization/Death	0 (0)	0 (0)	0 (0)	

452 Data are presented as n (%) unless otherwise indicated.
453 Abbreviations: IQR, interquartile range.
454 [a] ≥ 65 years old or comorbidity (obesity, diabetes, active cancer, chronic cardiovascular, pulmonary, liver, renal or inflammatory disease)
455 [b] Arrythmia, coronary disease.
456 [c] Chronic obstructive pulmonary disease, fibrosis.
457 [d] Stage III–V according to CKD classification

For peer review only

Table 4. Multivariate logistic regression for COVID diagnosis

RFE selection order	Feature groups: LUS findings (*n=10*)	Feature groups: Symptoms (*n=8*)	Feature groups: Vital signs (*n=3*)	Feature groups: Epidemiological history (*n=1*)	Coefficient*: Neg	Coefficient*: Pos
**1** *(removed last)*		Cough				0.40
**2**	Pleural thickening (any)					0.69
**3**	Pleural thickening (number of sites)				-0.40	
**4**		Fever				0.44
**5**	Confluent B lines (number of sites)					0.41
**6**	Normal pattern (number of sites)					0.29
**7**	Pathologic B lines (number of sites)					0.49
**8**		Anosmia				0.43
**9**				Contact with COVID-19		0.47
**10**		Dyspnea			-0.28	
**11**		Myalgia				0.37
**12**		Diarrhea			-0.49	
13	Multifocality				-0.26	
**14**		Rhinorrhea				0.35
**15**		Sputum				0.41
**16**			Oxygen saturation			0.20
**17**	Consolidation (any)				-0.18	
**18**			Temperature (°C)			0.22

Diagnostic performance with various feature sets:

	**22-0 features=22** *10 LUS, 8 symptoms, 1 contact, 3 signs*	**22-7 features=15** *6 LUS, 8 symptoms, 1 contact, **NO** signs*	**22-12 features=10** *5 LUS, 4 symptoms, 1 contact, **NO** signs*	**22-14 features=8** *5 LUS, 3 symptoms, **NO** contact, **NO** signs*
	Sens: 78.8%	Sens: 75.8%	Sens: 84.8%	Sens: 81.8%
	Spec: 84.0%	Spec:83.2%	Spec: 72.3%	Spec: 62.2%
	AUC: 84.5%	AUC: 83.5%	AUC: 80.2%	AUC: 76.6%
	LR+: 4.9	LR+: 4.5	LR+: 3.1	LR+: 2.2
	LR-: 0.3	LR-: 0.3	LR-: 0.2	LR-: 0.3
	PPV: 83.1%	PPV: 81.8%	PPV: 75.4%	PPV: 68.4%
	NPV: 61.4%	NPV: 80.6%	NPV: 73.5%	PPV: 64.7%

**LUS findings only**	**Clinical only**
Sens: 45.5%	Sens: 72.7%
Spec: 77.3%	Spec: 79.8%
AUC: 63.9%	AUC: 80.3%

**19**			Respiratory rate		-0.30			LR+: 2.0	LR+: 3.6
**20**	Consolidation (any)				-0.18			LR-: 0.7	LR-: 0.3
**21**	Pathologic B lines (any)				-0.07			PPV: 66.7%	PPV: 78.3%
**22** *(removed first)*	Confluent B lines (any)				0.26			NPV: 55.6%	NPV: 64.5%

Multivariate logistic regression for COVID diagnosis where selection order is indirectly proportional to the feature's predictive importance, in recursive feature elimination (RFE), i.e., the feature labeled 22 was removed first, while 1 was retained until the end. Four feature groups containing 10 LUS findings, 8 symptoms, 3 vital signs and 1 epidemiological history of contact are color-coded according to their coefficient in the multivariate score including all 22 features (orange positive correlation with COVID and blue negative correlation). *The coefficient in multivariate scores is susceptible to multicollinearity.

For peer review only

465 **List of Supplemental Digital Content**

466 SupplementaryTables.docx

For peer review only

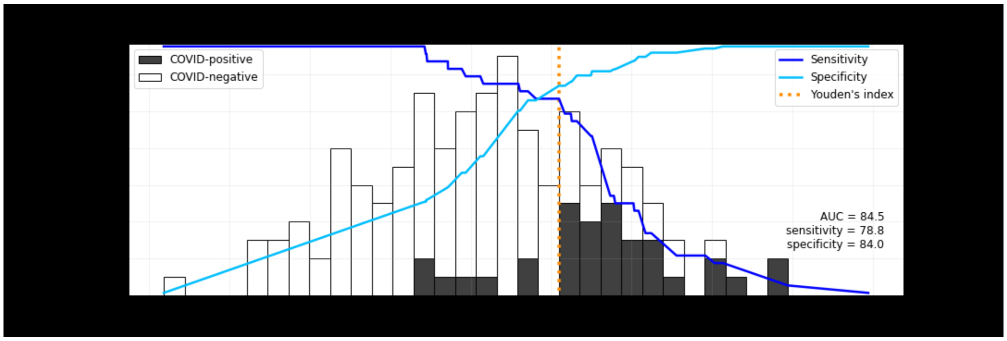

Figure 1. A multivariate logistic regression diagnostic score (x-axis) to discriminate COVIDpos from COVIDneg patients (black and white bars respectively with count on y axis). Sensitivity (—) and specificity (—) of the score are plotted with Youden's index (sensitivity + specificity -1) marked in orange. All 22 features are used in the depicted image on a model trained on all data points.

381x127mm (72 x 72 DPI)

Supplementary Tables.

Supplementary Table 1. Characteristics of study participants comparing healthy controls and patients with a lower respiratory tract infection ($COVID^{pos}$ and $COVID^{neg}$).

	All (n=178)	LRTI patients	Control patients	
Female sex	112 (63)	84 (63)	28 (64)	0.910
Age, years; Median (IQR)	34 [28, 45]	35 [29, 46]	31 [25, 42]	0.007
Pulmonary disease [a]	3 (1.7)	3 (2.2)	0 (0)	0.317
Current cigarettes smoker	51 (29)	39 (29)	12 (27)	0.816

Data are presented as n (%) unless otherwise indicated.
Missing values: 0
Abbreviations: IQR, interquartile range; LRTI, Lower respiratory tract infection
[a] COPD, fibrosis.

For peer review only

Supplementary Table 2. Lung ultrasound characteristics of study participants comparing healthy controls and patients with a lower respiratory tract infection (COVID^{pos} and COVID^{neg}).

	**All (n=178)**	**LRTI patients (n=134)**	**Control patients (n=44)**	
Abnormal lung ultrasound	45 (25)	41 (31)	4 (9.1)	0.004
Abnormal lung ultrasound apart from focal B lines	31 (17)	30 (22)	1 (2.2)	0.002
Multifocal	16 (9.0)	16 (12)	0 (0)	0.016
Bilateral	8 (4.5)	8 (6.0)	0 (0)	0.097
Number of pathologic zones; Median (IQR)	0 [0, 0.7]	0 [0, 1]	0 [0, 0]	0.003
Pathologic B lines (≥3)	23 (13)	20 (15)	3 (6.8)	0.164
Confluent B lines (White lung)	12 (6.7)	11 (8.2)	1 (2.3)	0.173
Thickening of the pleura with pleural line irregularities	18 (10)	18 (13)	0 (0)	0.010
Consolidations (>1cm)	1 (0.6)	1 (0.8)	0 (0)	0.566
Pleural effusion	0 (0)	0 (0)	0 (0)	
LUS score; Median (IQR)	0 [0, 0.75]	0 [0, 1]	0 [0, 0]	0.003

Data are presented as n (%) unless otherwise indicated.
Abbreviations: IQR, interquartile range.

Supplementary Table 3. Lung ultrasound characteristics of study participants comparing healthy controls and COVID-19 patients

For peer review only - http://bmjopen.bmj.com/site/about/guidelines.xhtml

	**All (n=75)**	**COVID-19 patients (n = 31)**	**Control patients (n = 44)**	
Abnormal lung ultrasound	18 (24.0)	14 (45)	4 (9.1)	0.001
Abnormal lung ultrasound apart from focal B lines	10 (13)	9 (29)	1 (2.2)	0.003
Multifocal	6 (8)	6 (19)	0 (0)	0.009
Bilateral	3 (4)	3 (9.7)	0 (0)	0.132
Number of pathologic zones; Median (IQR)	0 [0, 0]	0 [0, 1]	0 [0, 0]	< 0.001
Pathologic B lines (≥3)	9 (12)	6 (19)	3 (6.8)	0.199
Confluent B lines (White lung)	5 (6.7)	4 (13)	1 (2.3)	0.178
Thickening of the pleura with pleural line irregularities	6 (8)	6 (19)	0 (0.0)	0.009
Consolidations (>1cm)	0 (0)	0 (0)	0 (0)	
Pleural effusion	0 (0)	0 (0)	0 (0)	
LUS score; Median (IQR)	0 [0, 0]	0 [0, 2.5]	0 [0, 0]	<0.001

Data are presented as n (%) unless otherwise indicated.
Abbreviations: IQR, interquartile range.

For peer review only

STROBE Statement—Checklist of items that should be included in reports of ***cohort studies***

	Item No	Recommendation	Page No
**Title and abstract**	1	(*a*) Indicate the study's design with a commonly used term in the title or the abstract	1
		(*b*) Provide in the abstract an informative and balanced summary of what was done and what was found	2
**Introduction**			
Background/rationale	2	Explain the scientific background and rationale for the investigation being reported	4
Objectives	3	State specific objectives, including any prespecified hypotheses	4
**Methods**			
Study design	4	Present key elements of study design early in the paper	5
Setting	5	Describe the setting, locations, and relevant dates, including periods of recruitment, exposure, follow-up, and data collection	5
Participants	6	(*a*) Give the eligibility criteria, and the sources and methods of selection of participants. Describe methods of follow-up	5
		(*b*) For matched studies, give matching criteria and number of exposed and unexposed	
Variables	7	Clearly define all outcomes, exposures, predictors, potential confounders, and effect modifiers. Give diagnostic criteria, if applicable	7
Data sources/ measurement	8*	For each variable of interest, give sources of data and details of methods of assessment (measurement). Describe comparability of assessment methods if there is more than one group	8
Bias	9	Describe any efforts to address potential sources of bias	5
Study size	10	Explain how the study size was arrived at	6
Quantitative variables	11	Explain how quantitative variables were handled in the analyses. If applicable, describe which groupings were chosen and why	7
Statistical methods	12	(*a*) Describe all statistical methods, including those used to control for confounding	9
		(*b*) Describe any methods used to examine subgroups and interactions	7
		(*c*) Explain how missing data were addressed	9
		(*d*) If applicable, explain how loss to follow-up was addressed	N/A
		(*e*) Describe any sensitivity analyses	8
**Results**			
Participants	13*	(a) Report numbers of individuals at each stage of study—eg numbers potentially eligible, examined for eligibility, confirmed eligible, included in the study, completing follow-up, and analysed	9
		(b) Give reasons for non-participation at each stage	9
		(c) Consider use of a flow diagram	N/A
Descriptive data	14*	(a) Give characteristics of study participants (eg demographic, clinical, social) and information on exposures and potential confounders	9
		(b) Indicate number of participants with missing data for each variable of interest	21
		(c) Summarise follow-up time (eg, average and total amount)	11
Outcome data	15*	Report numbers of outcome events or summary measures over time	11

For peer review only - http://bmjopen.bmj.com/site/about/guidelines.xhtml

Main results	16	(*a*) Give unadjusted estimates and, if applicable, confounder-adjusted estimates and their precision (eg, 95% confidence interval). Make clear which confounders were adjusted for and why they were included<br>(*b*) Report category boundaries when continuous variables were categorized<br>(*c*) If relevant, consider translating estimates of relative risk into absolute risk for a meaningful time period	9
Other analyses	17	Report other analyses done—eg analyses of subgroups and interactions, and sensitivity analyses	10
**Discussion**			
Key results	18	Summarise key results with reference to study objectives	12
Limitations	19	Discuss limitations of the study, taking into account sources of potential bias or imprecision. Discuss both direction and magnitude of any potential bias	14
Interpretation	20	Give a cautious overall interpretation of results considering objectives, limitations, multiplicity of analyses, results from similar studies, and other relevant evidence	14
Generalisability	21	Discuss the generalisability (external validity) of the study results	14
**Other information**			
Funding	22	Give the source of funding and the role of the funders for the present study and, if applicable, for the original study on which the present article is based	15

*Give information separately for exposed and unexposed groups.

Note: An Explanation and Elaboration article discusses each checklist item and gives methodological background and published examples of transparent reporting. The STROBE checklist is best used in conjunction with this article (freely available on the Web sites of PLoS Medicine at http://www.plosmedicine.org/, Annals of Internal Medicine at http://www.annals.org/, and Epidemiology at http://www.epidem.com/). Information on the STROBE Initiative is available at http://www.strobe-statement.org.

For peer review only
